# Behavioral Tests in Neurotoxin-Induced Animal Models of Parkinson’s Disease

**DOI:** 10.3390/antiox9101007

**Published:** 2020-10-16

**Authors:** E. Maruthi Prasad, Shih-Ya Hung

**Affiliations:** 1 Graduate Institute of Acupuncture Science, College of Chinese Medicine, China Medical University, No.91, Hsueh-Shih Road, Taichung 40402, Taiwan; emaruthip@gmail.com; 2 Department of Medical Research, China Medical University Hospital, No. 2, Yude Road, Taichung 40447, Taiwan

**Keywords:** animal behavioral studies, motor symptoms, MPP^+^, MPTP, neurotoxins, paraquat, Parkinson’s disease, permethrin, rotenone, 6-OHDA

## Abstract

Currently, neurodegenerative diseases are a major cause of disability around the world. Parkinson’s disease (PD) is the second-leading cause of neurodegenerative disorder after Alzheimer’s disease. In PD, continuous loss of dopaminergic neurons in the substantia nigra causes dopamine depletion in the striatum, promotes the primary motor symptoms of resting tremor, bradykinesia, muscle rigidity, and postural instability. The risk factors of PD comprise environmental toxins, drugs, pesticides, brain microtrauma, focal cerebrovascular injury, aging, and hereditary defects. The pathologic features of PD include impaired protein homeostasis, mitochondrial dysfunction, nitric oxide, and neuroinflammation, but the interaction of these factors contributing to PD is not fully understood. In neurotoxin-induced PD models, neurotoxins, for instance, 6-hydroxydopamine (6-OHDA), 1-Methyl-4-phenyl-1,2,3,6-tetrahydropyridine (MPTP), 1-Methyl-4-phenylpyridinium (MPP^+^), paraquat, rotenone, and permethrin mainly impair the mitochondrial respiratory chain, activate microglia, and generate reactive oxygen species to induce autooxidation and dopaminergic neuronal apoptosis. Since no current treatment can cure PD, using a suitable PD animal model to evaluate PD motor symptoms’ treatment efficacy and identify therapeutic targets and drugs are still needed. Hence, the present review focuses on the latest scientific developments in different neurotoxin-induced PD animal models with their mechanisms of pathogenesis and evaluation methods of PD motor symptoms.

## 1. Background

Parkinson’s disease (PD) is an age-linked, advanced neuro-deteriorating illness primarily characterized by the loss of nigrostriatal dopaminergic neurons [1]. Parkinsonism prevalence is more than 1% of 55-year-olds and more than 3% of 75 years or older individuals in Europe [2]. The average number of All ethnic groups’ occurrence of PD is 13.4/100,000 individuals, wherein males and females the prevalence is 19/100,000 and 9.9/100,000 individuals, respectively [3]. In the United States, an estimated number of PD patients will be around one million by 2020; these numbers are projected to 1.06 million by 2030 and 1.24 million by 2040 [4,5]. PD is typically sporadic [6]. The age of PD onset varies widely. Patients who have been diagnosed with PD between 20 and 50 years old are categorized as early-onset PD. After 50 years, patients are categorized as late-onset PD [2]. The early forms of PD are often inherited (not always), some of which are related to specific genetic mutations [7]. A study of 3952 various ethnic background PD patients shows that 15.5% of cases are familial PD and 4.3% of cases are sporadic early-onset PD [8]. In the nigrostriatal pathway, the neurotransmitter dopamine transfers from substantia nigra (localized to midbrain) to the caudate nucleus and putamen, which is located in the dorsal striatum [9]. The damage of dopaminergic neurons prompts impaired afferents (excitatory or inhibitory) to the striatum and further leads to nerve terminal degeneration in the striatum and causes PD motor symptoms [10]. Nitric oxide also acts as a neurotransmitter and is a component of the signaling pathways that operate between cerebral blood vessels, neurons, and glial cells [11]. Increasing evidence shows that nitric oxide modulates neurotoxin-induced cell damage and is involved in neuronal cell death in PD [12].

Pathologically the PD is also known as a mitochondrial disease of senescence [13,14]. Additionally, hereditary factors and protein aggregation are associated with neurodegeneration of dopaminergic neurons in PD [15]. Alterations of genes such as *DJ-1*, *PINK1,* and *leucine-rich repeat kinase-2* (*LRRK2*) affect the mitophagy that impairs the mitochondrial respiratory chain and produces reactive oxygen species in the brain, which is associated with mitochondria dysfunction, dopaminergic neuronal death, and abnormal protein aggregation [16]. During the development of PD, α-synuclein accumulation has become more common in the brain [17,18]. Current PD treatment mainly targets motor symptom improvement; the drugs are designed to restore dopamine levels in the striatum or act on the striatum synaptic dopamine receptors [19]. Levodopa (a dopamine precursor) is an effective and well-tolerated dopamine replacement agent that has been used for over 50 years to improve motor symptoms [20]. Unfortunately, although levodopa therapy may initially improve PD’s motor symptoms, the benefits frequently wear off over time or become less consistent [20]. To date, no treatment is available to stop or slow the progression of PD [13,21]. New drug/treatment discovery for PD treatment is needed.

**Figure 1:**
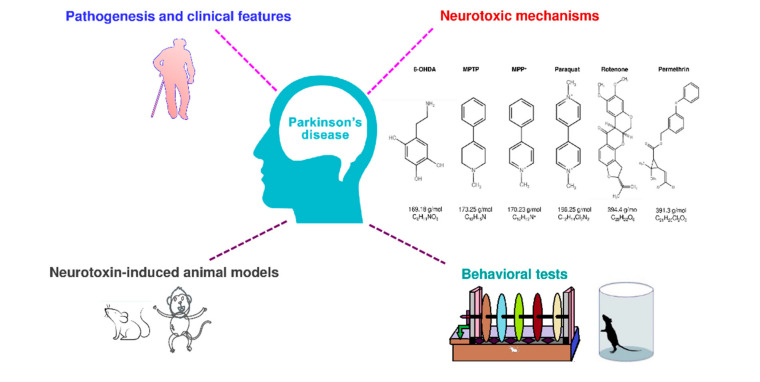
Graphic representation of pathogenesis, neurotoxic mechanisms, neurotoxin-induced animal models, and their behavioral tests.

Disease animal models are essential for drug/treatment discovery. An ideal animal model of PD should mimic all the clinical and pathologic characteristics of human PD. However, no currently used PD animal model can reproduce all the behavioral and pathologic features that have been seen in the typical form of human PD. Many studies show that specific neurotoxins can induce PD-like symptoms, including motor defects, progressive loss of dopaminergic neurons in substantia nigra pars compacta, and Lewy bodies [22]. Although the effect of current treatments in these neurotoxin-induced animal models is not efficient in translating into clinical use, we think that the behavioral phenotyping in neurotoxin-induced PD animal models leads to effective treatment for PD in the future. In the present review, we have described pathogenesis, symptoms, and neurotoxin in PD, neurotoxin-induced PD animal models, and their behavioral evaluating methods from the recent five years’ literature (Figure 1).

## 2. Pathogenesis and Symptoms of PD

**Figure 2:**
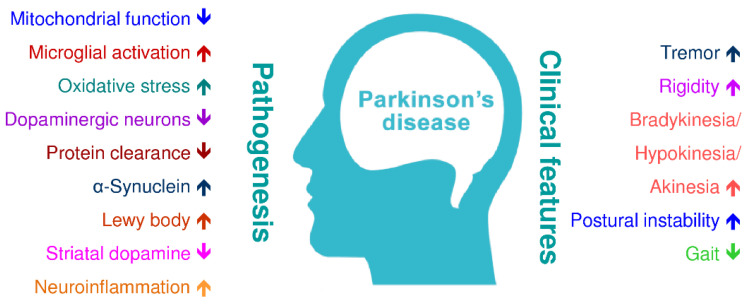
Pathogenesis and clinical features of Parkinson’s disease. Diagram illustrates the neurodegenerative changes leading to dopaminergic neuronal death, pathogenesis, and the clinical features of Parkinson’s disease.

Morphologic alterations of the PD brain show the disappearance of the dark-pigmented region of the substantia nigra [23,24]. This loss of pigmentation is directly proportional to the loss/decrease of dopamine neuro-melanin neurons in the substantia nigra [25]. At the onset of clinical symptoms, it is estimated that the loss of nigral dopaminergic neurons is up to 60% or more; it is closely related to the severity of motor functions and the duration of disease [26,27]. The consequence of neuronal death of dopaminergic neurons leads to denervation of the nigrostriatal pathway, which leads to a decrease of dopamine in the striatum, and the reduction in dopamine signals causes the primary motor symptoms in PD [28]. A comprehensive approach to understanding the pathogenesis of the PD can help identify and develop new therapies that can improve the PD treatment and control the clinical manifestations of the disease. Figure 2 illustrates the pathogenesis, possible toxic mechanisms, and clinical features of PD.

### 2.1. α-Synuclein Misfolding and Aggregation

In the substantial nigra of PD patients, the abnormal protein aggregates that progress and accumulate in nerves are Lewy bodies [29]. A presynaptic neuronal protein α-synuclein accumulates in Lewy bodies of neurons exhibit neurotoxic effects associated with PD’s pathogenesis [30,31,32]. Numerous mechanisms for α-synuclein abnormal conformational changes aggregation have been proposed, including phosphorylation of serine-129, ubiquitination, and C-terminal truncation [33,34]. As a result, different α-synuclein accumulate in the PD brain, including unfolded monomers, soluble oligomers, protofibrils, and high molecular weight insoluble fibrils [35,36,37]. Recent data from rodents suggested that the utmost neurotoxic α-synuclein are primary oligomeric forms rather than developed insoluble fibrils [38,39,40].

### 2.2. Impaired Functioning of Mitochondria

The loss of mitochondrial function is considered as a key factor in the PD prognosis—both idiopathic and familial [14]. Previous studies of autopsy in the substantia nigra of PD brains reported a lack of the mitochondrial complex I, which provided one of the foremost direct relations between mitochondrial dysfunction and PD [41]. 1-Methyl-4-phenyl-1,2,3,6-tetrahydropyridine (MPTP) is a lipid-soluble neurotoxin that can pass the blood-brain barrier and oxidized to its toxic metabolite 1-Methyl-4-phenylpyridinium (MPP^+^) by monoamine oxidase-B from astrocytes in both primate and rodent [42]. Then MPP^+^ is selectively up-taken by dopamine transporters on dopamine neurons to induce specific dopaminergic neuronal death [42]. After MPTP or MPP^+^ injection, the inhibition of mitochondrial complex I in dopaminergic neurons causes neuronal death and physiological and behavioral deficits, which leads to aggregation of α-synuclein-containing Lewy bodies directing to the production of reactive oxygen species [43]. Toxins and pesticides that inhibit mitochondrial complex I, such as rotenone and paraquat, also the reason for Parkinsonian phenotype and dopaminergic neuronal death [44]. The mitochondrial complex I dysfunction causes dopaminergic energy depletion and cell death [45]. An additional major clue to the role of mitochondria in the pathogenesis of PD is that many known genes leading to familial PD affect the homeostasis of mitochondria [46,47].

Mitophagy is the selective autophagic degradation of mitochondria; Lin et al. (2020) observed that MPP^+^ or MPTP-induced dopaminergic neuronal death, mitochondrial membrane polarization, ATP diminishing which was associated with mitophagy inhibition in SH-SY5Y cells [13]. For instance, *PINK1* (*PARK2*) and *PARKIN* (*PARK6*) regulate mitochondrial function and remove damaged mitochondria by activating mitophagy [48,49]. Mutational changes in the gene lead to diminished mitochondrial quality control and autosomal recessive PD [50,51]. Moreover, α-synuclein interferes with mitochondrial functions; for instance, it can react with mitochondrial membrane and aggregate in organelles [52]. α-synuclein prompts the damage of mitochondrial complex I roles and results in mitochondrial dysfunction and higher oxidative stress [53,54]. Di Maio et al. (2016) reported the interaction between oligomeric α-synuclein and mitochondrial receptor TOM20 (translocase of outer membrane) damaged the mitochondrial protein importation machinery, diminished respiration, and directed to excessive reactive oxygen species production [55].

### 2.3. Dysregulation of Protein Clearance Control

PD is linked with protein aggregation in dopaminergic neurons [56]. The increased protein aggregation can affect not only protein production but also impairs protein clearance. Cells have two major protein eliminating systems to remove dysfunctional proteins; they are ubiquitin-protease and autophagy-lysosome systems [57]. The ubiquitin-protease system’s primary function is to break unusual proteins by labeling with ubiquitin and transporting to the proteasome for degradation [58]. The disturbances of the ubiquitin-protease system contribute to the pathogenesis of both familial and sporadic PD [59]. The autophagy-lysosome system is subcategorized to macroautophagy, microautophagy, and chaperone-mediated autophagy [60,61]. Autophagy prevents the aggregation of proteins and impaired organelles; however, the imbalanced autophagy induction produces neurodegeneration. The impairment of the autophagy-lysosome system is progressively observed as a key pathogenic mechanism in PD [62]. According to Lin et al. (2020), the mechanism of celastrol against dopaminergic neuronal death was via increasing autophagosome formation and removing dysfunctional mitochondria by autophagy [13]. It suggests that autophagy activation is one of the important targets in PD treatment. The α-synuclein monomer is usually cleared by both the ubiquitin-protease system and the autophagy-lysosome pathways in the pathogenesis of PD by stimulating the aggregation of defective proteins, particularly soluble misfolded α-synuclein [63,64]. All these studies show that dysfunction of protein turnover can result in neuronal cell death, thus providing a potential PD pathogenic mechanism.

### 2.4. Neuroinflammation

The signs and symptoms of PD are specific and sometimes differ from patient to patient [65]. Premature PD signs may be slight and unnoticed [66]. The PD symptoms generally start on one side and begin to affect both sides of the body, then become massive (Figure 2) [67]. Neuroinflammation is one of the utmost significant events associated with the pathogenesis of PD [68]. Also, the PD inflammatory responses involve in both glial cell activation and peripheral immune cell infiltration, but the connection between the two diverse inflammatory pathways remains not fully understood [69]. These problems greatly reduced the development of PD therapies by targeting inflammatory pathways. Previous autopsy studies also showed the involvement of innate and adaptive immune responses in the brain of PD patients [70]. In a neurotoxin-induced PD rat model, Hung et al. (2008) showed that MPP^+^-induced neuroinflammation via increasing the production of TNF-α, IL-1β, in the substantia nigra and decreasing the striatal dopamine [71]. Previous studies conducted in several PD animal models showed that inflammatory processes play a chief role in dopaminergic neuronal cell death [72]. The neuroinflammatory process may represent the target of neuroprotection in PD; the different forms of microglial activation promote the increase in the difficulty and the complexity of manipulating microglia responses in PD [69,73].

## 3. Neurotoxins Used to Induce PD In Vivo Models

**Figure 3:**
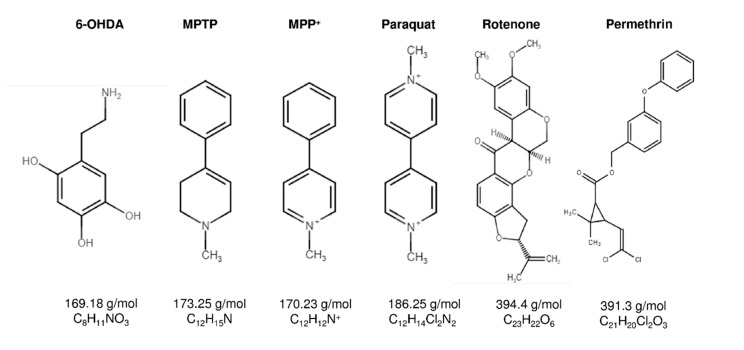
Chemical structures of neurotoxins used to induce Parkinsonism in animal models.

The pharmacological diseased animal models of PD are essential tools in the investigation of therapeutics. There are several neurotoxins, which are the structural analog of catecholamine, dopamine, and noradrenaline. The recent studies applied the neurotoxins to induce the PD, such as 6-hydroxydopamine (6-OHDA), MPTP, MPP^+^, paraquat, rotenone, and permethrin (Figure 3). The lesioning types and induction methods to produce PD animal models are different; each neurotoxin has its specific destructive pathways induce brain neurodegeneration (Table 1).

### 3.1. 6-OHDA

**Figure 4:**
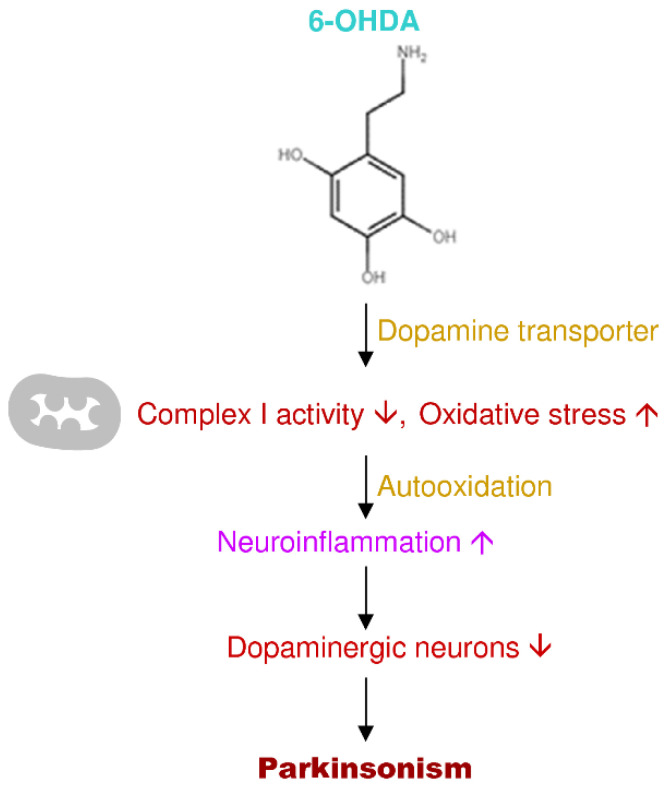
Putative mechanism of action of 6-hydroxydopamine (6-OHDA) in PD progression.

The neurotoxin 6-OHDA is one of the proposed for modeling PD research in animal models [74]. The administration of 6-OHDA is variably unilateral into the nigrostriatal pathway to produce neurodegeneration, and various 6-OHDA animal models have been conventional for the PD investigations [75,76]. 6-OHDA is a lethal toxin that mainly damages peripheral and central nervous systems [77]. Nevertheless, the neurotoxin 6-OHDA cannot pass the blood-brain barrier; the central nervous system toxicity obtains only by direct administration into the brain [78]. The toxic effects of 6-OHDA happen through a twofold mechanism involving toxins aggregating into catecholaminergic neurons and then modifying cell steadiness and neuronal destruction [79]. Dopamine or norepinephrine membrane transport protein uptake 6-OHDA because it is similar to the structure of endogenous catecholamine [80]. The toxicity of 6-OHDA is related to its ability to the production of free radicals and oxidative stress, which is similar to the hydrogen peroxide effect [81]. The inhibition of brain mitochondrial complexes (I and IV) and oxidation to form semiquinone radicals (which participate in reactive oxygen species production) are the main biochemical properties of 6-OHDA. Then the toxicity results in the loss of respiratory activity from oxidative stress by free radicals [82,83]. Rodriguez-Pallares et al. (2007) data showed that the autooxidation-derived oxidative stress and the inhibition of the mitochondrial respiratory chain, initially microglial stimulation and NADPH oxidase-derived reactive oxygen species functions synthetically with 6-OHDA and establish an appropriate and early constituent of 6-OHDA-convinced cell death (Figure 4) [84].

Liu et al. (2018) administered 6-OHDA at 4 µg/µL in saline and ascorbic acid (0.02%) at a rate of 1 µL/min to induce PD in male Sprague Dawley rats. They have assessed the effect of 6-OHDA-induced motor effects using apomorphine (0.5 mg/kg, subcutaneously) injection after the two weeks of surgery for the rotational behavior and reported that olfactory ensheathing cells possess neuroprotective effect in 6-OHDA-induced rat models [85]. Boix et al. (2015) administered four different doses, such as 0.3, 0.7, 1.0, and 3.6 µg/µL of 6-OHDA (in 0.02% ascorbic acid and saline) unilaterally into midforebrain to induce the neurodegeneration and established a 6-OHDA animal model in male Rgs5^gfp/+^ reporter mice. They assessed the behavioral changes for the four different doses of PD model mice through corridor test (three weeks’ post-lesion), cylinder test (five weeks after the lesion), stepping test (five weeks after lesion), apomorphine-induced (0.1 mg/kg, subcutaneously) rotational test, and amphetamine-induced rotational test (5 mg/kg, intraperitoneally five weeks after the lesion). According to the behavioral tests of the corridor, amphetamine-induced rotational, stepping, and cylinder, they revealed significant changes; where the spontaneous rotation and apomorphine-induced rotational tests not displayed considerable results with the partial midforebrain lesion [86]. Jalewa et al. (2017) injected 5 µg of 6-OHDA in 5 µL at a speed of 1 µL/min into the right midforebrain in male Sprague Dawley rats. The 6-OHDA injury-induced behavior tests stressed by apomorphine (0.05 mg/kg, subcutaneously) or amphetamine (3 mg/kg, intraperitoneally) injections by regulating dopaminergic transmission. Their data revealed that the dual agonist GLP-1R/GIPR receptor dual agonist DA–JC1 controlled the circling behavior, representing that dopaminergic transmission was improved by the drug. Their data for DA–JC1 also showed an impact on the rotarod and open-field tests [87]. Smith et al. (2016) used 6-OHDA at a dose of 6 µg in 3 µL (0.3 µL/min infusion rate) injected into the left midforebrain unilaterally in male Wistar rats. They examined the 6-OHDA lesion range by assessing the adjusting step test and the forelimb use asymmetry test on the 5th day after the lesion and found that lesioned rats affected with a right paw for less than 5% of movements in both the step test and the asymmetry test [88]. Ribeiro et al. (2016) showed that the low dose of 6-OHDA (40 µg/site, intracerebroventricular injection) affect the striatal biochemical parameters that correlated to oxidative stress and produced forelimb inability in male Swiss mice [89]. Their experiments showed that 6-OHDA dose (40 µg/site) exhibited neither hypokinesia nor reduced forelimb strength, produced bradykinesia, and the succinobucol restored the bradykinesia by decreasing the forelimb ability without changes in locomotion [89]. Lai et al. (2019) used three doses of 5/10/15 µg of 6-OHDA in 4 µL (in saline) and intracranially injected into right midforebrain injection to induce the lesion in male Sprague Dawley rats. The behavioral outcomes showed an improvement in the rotarod retention time in 10 µg/4 µL group at 119 s staying time and vehicle-treated group at 170 s staying time in the valproate-treated group. Valproate failed to rescue the rotarod performance at the 6-OHDA dose of 5 µg and 15 µg groups dopamine neurodegeneration. Their data showed that valproate (100 mg/kg, for two weeks) protected from the loss of dopaminergic neurons and increased motor performance in 6-OHDA rats compared to the normal control group rats [90]. Vieira et al. (2019) administered 6 µg of 6-OHDA in 1 µL of artificial cerebrospinal fluid (at 0.33 µL/min) injected intranigrally to generate the PD-like lesion in male Wistar rats. The 6-OHDA administration also prompted anxiety-like behavior in the elevated plus-maze and contextual fear conditioning tests conducted on the 21st and 24th day, respectively [91]. Their neurochemical results also proved that the anxiety might be related to a monoamines dysregulation in the amygdala, prefrontal cortex, and striatum in PD [91]. Penttinen et al. (2016) administered a low dose of 6-OHDA, i.e., 6 µg (3 sites × 2 µg) equally to three sites into the striatum at the rate of 0.5 µL/min in male Wistar rats. The administrated rats followed by amphetamine-induced (2.5 mg/kg, subcutaneously) rotations and showed consistent rotational behavior and affected striatal tyrosine hydroxylase-positive loss and dopamine transporter-positive neurites. Their data for the rotational behavioral changes during the 1st–2nd week without significant changes, the motor asymmetry revealed only during the 8th–14th, suggested that the low dose 6-OHDA (6 µg) rats showed stable motor impairments and suitable for neurological therapeutic investigations [92]. Quiroga-Varela et al. (2017) developed a rodent model of PD by using the intracerebroventricular injection of 6-OHDA at the doses of 100, 300, 500, 700, and 1000 µg in 4 µL (at 1 µL/min, 1st–10th day) to achieve bilateral and progressive motor alterations associated with progressive nigrostriatal dopaminergic degeneration in male Sprague Dawley rats. The experimental study used repeated intracerebroventricular 6-OHDA dose models to alter the bilateral and progressive motor behaviors related to progressive degeneration nigrostriatal dopaminergic pathways [93]. The injection of a single 6-OHDA infusion (100 µg) caused an increase in catalepsy compared to other experimental groups [93]. Oliveira-Giacomelli et al. (2019) tested the effects of P2Y6 and P2X7 in preventing or reversing PD behavior and dopaminergic levels in male Sprague Dawley rat models using 7 µg/µL of 6-OHDA dose injected unilaterally into the right midforebrain of two-month-old male Sprague Dawley rats. The motor changes confirmed after the 1st week of 6-OHDA lesion, behavioral analysis data showed no significant rotational behavior before 6-OHDA lesion, and significant behavioral changes were observed only after 1st, 3rd, and 5th week of 6-OHDA lesion where the number of rotations which increased in a time-dependent manner [94].

Park et al. (2018) administered 0.5, 1, 2, and 4 µg/µL of 6-OHDA in 1 µL (in saline and ascorbic acid at 0.5 µL/min) injected unilaterally into the right midforebrain to induce the PD in C57BL/6 mice models. The 4 µg/µL high dose 6-OHDA group mice data revealed significantly worsen impairment in forelimb through wall contact compared to control mice. The subthalamic nucleus firing rate showed considerably high in groups with approximately 75% dopamine-producing cell damage in the substantia nigra, but a slight rise was detected in partial lesion groups compared to the normal control group. Electrophysiology data showed that the firing patterns for high dose were irregular and revealed burst-like patterns with dominant slow-wave oscillations in between the 0.3–2.5-hertz frequency range; the study outcomes demonstrated a relationship between neurological activities in the subthalamic nucleus and dopamine exhaustion in the nigrostriatal pathway influenced by dose differences of 6-OHDA [95]. Guimarães Marques et al. (2019) administered 6, 12, and 24 µg doses of 6-OHDA (in 0.5 µL) model of *Proechimys* for investigating the mechanisms of the vulnerability of dopaminergic neurons loss during the PD. Their studies revealed standard investigative behavior throughout the cylinder test in all animals, observed no significant changes in the tyrosine hydroxylase expression levels in both striatum and substantia nigra, and proposed that *Proechimys* were unaffected to dopaminergic neuronal deaths [96]. Sarookhani et al. (2018) administered 4 µL (4 µg/µL) of 6-OHDA unilaterally into two sites and examined the sodium hydrosulfate (5.6 mg/kg) activity in male Wistar rat models. They used apomorphine (0.5 mg/kg, intraperitoneally) before the behavioral tests and found the motor defects contralateral rotations in all 6-OHDA-induced groups, revealed that pretreatment with sodium hydrosulfate did not protect 6-OHDA-induced PD impairments [97]. Romero-Sánchez et al. (2020) administered 6, 10, and 16 µg of 6-OHDA in 2 µL (at 0.1 µL/min) unilaterally into left substantia nigra to induce the lesion and revealed that the impaired nigrostriatal dopaminergic pathway in male Wistar rat models. The 6-OHDA (16 µg dose) unilateral lesion affected the animal as a neurotoxic model of PD; i.e., the painful sensation, for instance, caused the allodynia and hyperalgesia [98]. Besides, a regular pramipexole (3.0 mg/kg, intraperitoneally) treatment prevented the allodynia and hyperalgesia-induced by 6-OHDA in PD model rats [98]. Huang et al. (2019) administered 8 µg of 6-OHDA dose in 4 µL (saline and ascorbic acid) and applied unilateral injection to induce PD symptoms in male Sprague Dawley rat models. Their data for the behavioral tests (rotarod test, open-field test, and grid test), the resveratrol (30 mg/kg) considerably relieved 6-OHDA-induced motor dysfunction [99]. Mishra and Krishnamurthy. (2019) revealed the influence of rebamipide (20, 40, and 80 mg/kg- oral ingestions twice per day; i.e., 4th–27th day) against 6-OHDA-lesioned (20 µg 6-OHDA in 4 µL at 1 µL/min) Parkinsonian in the male Charles Foster strain albino rats. The rebamipide repressed 6-OHDA-lesioned motor impairments using behavioral tests such as apomorphine-induced (1 mg/kg, intraperitoneally) head rotation test, open-field test, rotarod test, grip strength test, and bar catalepsy test, and also the α-synuclein aggregations in substantia nigra [100]. Lima et al. (2017) revealed the neuroprotective effect of *Spirulina platensis* using 6 µg 6-OHDA in 1 µL-induced lesion in male Wistar rats. The *S. platensis* protected the reduction of apomorphine-induced (3 mg/kg, subcutaneously) rotational behavior associated with the 6-OHDA-lesioned group. The 6-OHDA group showed higher the number (250-fold) of contralateral rotations/h compared to other experimental groups [101]. Szot et al. (2016) administered 5, 10, and 14 µg/µL of 6-OHDA dose and injected bilaterally to obtain the neurodegenerations in male C57BL/6 mice. The 5 µg/µL dose of 6-OHDA consistently caused a significant abnormality in immobility time than other experimental mice groups. The behavioral test data for a forced swim (during 3rd week) and sucrose consumption test (during the 4th and 3rd week after the lesion) showed depressive-like behavior of locus coeruleus in 6-OHDA (5 µg/µL) mice [102]. Kamińska et al. (2017) unilaterally injected 6-OHDA at the dose of 8, 12, 16 µg/4 µL (at 0.5 mL/min) into brain midforebrain in male Wistar Han rats and studied the motor impairments using rotational behavior (apomorphine 0.25 mg/kg, subcutaneously) and a sucrose preference test. The 16 µg dose of 6-OHDA diminished the sucrose solution preference for 3% both in rats without and with desipramine pretreatment. Besides, 6-OHDA doses also affected the reduction of dopamine contents of brain structures on the ipsilateral side [103]. Haddadi et al. (2015) administered 6-OHDA (8 µg/2 µL) unilaterally into the substantia nigra in male Wistar rats and assessed the catalepsy (bar test) and motor coordination (rotarod test) behavioral tests. Their data revealed a significantly higher value in a catalepsy of 6-OHDA-induced rats whereas; in silymarin (100, 200, and 300 mg/kg, intraperitoneally five days) treated rats’ catalepsy reduced and observed significant impairments of motor disabilities in 6-OHDA group rats [104].

Haddadi et al. (2018) revealed the pretreatment benefits of gastrodin in 6-OHDA unilaterally lesioned (8 µg/2 µL/rat at 0.2 µL/min) male Wistar rats. Their data of 6-OHDA-induced motor imbalances by catalepsy test and rotarod test revealed the gastrodin pretreatment (20, 40, and 80 µg/3 µL/rat for five consecutive days) improved motor performances in a dose-dependent manner [105]. Kwan et al. (2020) administered unilaterally 17.5 µg of 6-OHDA into the right midforebrain to induced PD-like symptoms in female Sprague Dawley rats. They tested the abnormal involuntary movements caused by 6-OHDA on ondansetron (100 and 1000 ng/kg subcutaneously, 22 days) or vehicle in or with the levodopa, following which the influence of ondansetron in PD. Their results showed that the ondansetron at 100 ng/kg to levodopa caused a substantial decrease in motor impairments severity when compared to control and experimental groups. The ondansetron (100 ng/kg) simultaneously with levodopa reduced the progression of abnormal involuntary movements; it showed less severity compared to levodopa/vehicle groups [106]. Gomes et al. (2019) given 20 µg of 6-OHDA in 3 µL (in saline and ascorbic acid) bilaterally into dorsolateral striatum to induce the neurodegeneration in male Wistar rats and assessed the motor and metabolic outcomes on the 7th, 21st, or 35th day after 6-OHDA lesion. The motor abnormalities paralleled without a noteworthy change in body mass, food consumption, glucose tolerance, insulin sensitivity, and other biochemical parameters (such as plasma insulin levels, triacylglycerol, and total cholesterol levels) [107]. Voronin et al. 2019 in their study, administered 5 µg of 6-OHDA in 1 µL (saline and ascorbic acid at 0.5 µL/min) intrastriatal (into right striatum) and evaluated the effect of afobazole (2.5 mg/kg, intraperitoneally) in 6-OHDA-lesioned male CD-1 mice model. The 6-OHDA administration decreased the striatal dopamine content, which is closely related to tyrosine hydroxylase-positive neuronal death in the substantia nigra and lower the latency in the rotarod test [108]. Konieczny et al. (2017) administered 8 µg of 6-OHDA in 2 µL unilaterally injected to induce the PD symptoms and to evaluate lactacystin (2.5 µg/2 µL) neurological therapeutic activity in male Wistar rats. They applied the amphetamine (5 mg/kg, subcutaneously) or apomorphine-induced (0.25–0.75 mg/kg, subcutaneously) for rotational behavior in automated rotameters during the 1st, 3rd, and 6th week after 6-OHDA lesion. They found similar lactacystin effects (such as loss of striatal and nigral dopamine), reaching subsequently six weeks post-lesion; the deterioration comparable to that created by 6-OHDA caused supersensitivity of dopamine D2 without affecting D1 receptors in the striatum of 6-OHDA lesion [109]. Yu et al. (2018) administered 8 µg of 6-OHDA unilaterally into the left midforebrain in male Sprague Dawley rats, and they proved that the glucose-dependent insulinotropic polypeptide injections stabilized apomorphine-induced (0.5 mg/kg, subcutaneously) rotational behavior and revealed the positive changes in open-field test and anxiety-like behaviors [110]. Liu et al. (2019) administered 20 µg of 6-OHDA in 4 µL (at 0.5 µL/min) unilateral injection into the midforebrain to induce the neurodegenerative lesion in male Sprague Dawley rats. They studied the effect of dextromethorphan (20 mg/kg twice daily, intraperitoneally) administration from seven days before the 6-OHDA-induced lesion up to 28 days after the lesion. In the apomorphine-induced (0.2 mg/kg, subcutaneously) rotational test for the 6-OHDA group, the average contralateral rotations showed 10 turns/h before 6-OHDA lesion incidence, while the average number improved significantly to 527.8.6 ± 356.4 turns/h at the end of 4th week [111]. Su et al. (2017) administered 20 µg of 6-OHDA (in 4 µL, 2 µL/for each site at 0.2 µL/min) unilaterally into the left striatum and analyzed the effect of (5R)-5-hydroxytriptolide in male Sprague Dawley rats. Oral ingestion of (5R)-5-hydroxytriptolide (31.25, 62.5 and 125 µg/kg for five weeks) significantly alleviated apomorphine-induced (0.5 mg/kg, subcutaneously) rotations at a high dose of 125 µg/kg developed the behavioral performance compared to low dose or other experimental groups [112]. Zhang et al. (2018) administered 6-OHDA at the dose of 16 µg/ 4µL (saline and ascorbic acid) unilaterally in the right midforebrain in female Sprague Dawley rat models. Chronic levodopa treatment (25 mg/kg twice a day for three weeks, intraperitoneally) and benserazide (6.25 mg/kg/day, intraperitoneally) revealed the abnormal involuntary movements for the dyskinetic symptoms (abnormal involuntary movements and forelimb functional test). Their results showed that the 6-OHDA-lesioned rats treated with α-lipoic acid (31.5 or 63 mg/kg for three weeks) plus levodopa reduced the levodopa-induced dyskinesia dose-dependently [113]. Lee et al. (2019) administered 8 µg of 6-OHDA in 3 µL of sterile saline intracranially in male Sprague Dawley rat models. They applied behavioral tests such as elevated body swing testing, beam walk, apomorphine-induced (0.3 mg/kg, subcutaneously) contralateral rotations, and rotarod test during the 14th–56th day post-6-OHDA lesion. The human umbilical cord blood and plasma-treated groups showed a significant reduction of the motor impairments, where their outcomes also demonstrated the improved gut motility and dopaminergic neuronal survival of substantia nigra [114].

Huang et al. (2018) administered 16 µg of 6-OHDA (dissolved in 8 µL of saline and ascorbic acid) unilaterally into the right midforebrain in male Sprague Dawley rats. They evaluated the effect of metabotropic glutamate receptor-5-specific antagonist 2-methyl-6-(phenylethynyl) pyridine on levodopa-induced dyskinesia and the synaptic activity at the striatum of 6-OHDA-lesioned rats. The 2-methyl-6-(phenylethynyl) pyridine increased perforated synapses ratio by levodopa, while the combined administration of levodopa and 2-methyl-6-(phenylethynyl) pyridine restored the postsynaptic response effect [115]. Crabbé et al. (2018) administered 24 µg of 6-OHDA in 4 µL (in 0.05% ascorbate saline) unilaterally in female Wistar rats. The 6-OHDA injection impaired behavioral performances of PD rats. They showed that the metabotropic glutamate receptor-5-binding potential did not affect the levodopa treatment [116]. Sampaio et al. 2019 administered 5, 10, or 20 µg of 6-OHDA (in 2 µL at 1 µL/min) bilaterally in male Wistar rats. They evaluated the behavioral tests using open-field, rotarod, olfactory discrimination, object recognition, step-down inhibitory avoidance, forced swim, elevated plus maze, and the sucrose preference tests on the 7th, 21st, or 42nd day after 6-OHDA lesion. The 6-OHDA dose at 20 µg/hemisphere-induced neuronal death in the locus coeruleus and disrupted the motor function, whereas 6-OHDA dose at 5 µg/hemisphere-induced the short-term memory shortfalls in all experimental periods after 6-OHDA lesion [117]. Zhang et al. (2017) administered 8 µg/4 µL of 6-OHDA unilaterally into the right substantia nigra in male Sprague Dawley rats. They evaluated the effect of echinacoside (3.5 or 7 mg/kg, once a day for 14 days, intraperitoneally) against 6-OHDA lesioned rats. Apomorphine (0.5 mg/kg, intraperitoneally)-induced rotational test for 6-OHDA group increased contralateral rotations compared to control and experimental groups, no significant modifications between the 6-OHDA group and the 6-OHDA plus echinacoside group rats, but during the 5th week, post-6-OHDA plus echinacoside (3.5 and 7 mg/kg) group exhibited a significantly reduced rotation number [118]. Vaz et al. (2020) administered 10 µg of 6-OHDA intrastriatal (at 0.5 µL/min) in male CD1 wild-type mice and assessed the effect of tapentadol (2.8 and 5.0 mg/kg, intraperitoneally) against 6-OHDA-induced lesion. Behavioral tests such as the cylinder, abnormal involuntary movement ratings, and gait tests showed abnormal motor functions due to 6-OHDA-induced Parkinsonian in rats and suggested that the tapentadol combined with levodopa prevented the worsening of abnormal involuntary movements functional test [119]. Manouchehrabadi et al. (2020) administered 6-OHDA (6 µg in 2.5 µL) intrastriatal in male Wistar rats and assessed the effect of carvacrol (10, 15, and 20 mg/kg for two weeks, intraperitoneally) against 6-OHDA-induced lesion. They applied behavioral tests such as apomorphine-induced (1 mg/kg in 0.5% ascorbic acid–saline, intraperitoneally) rotational test, pole test, catalepsy test, beam walking, rotarod test, and open-field tests (during 5th–15th day of experimental period) [120]. They revealed that the post-treatment with carvacrol (15 and 20 mg/kg) protected against motor impairments due to 6-OHDA administration [120]. Ren et al. (2016) administered 12 µg of 6-OHDA (in 6 µL saline with 0.02% ascorbate) unilaterally at the rate of 1 µL/min into the brain in male Sprague Dawley rats. Three weeks after 6-OHDA lesion, they administered the apomorphine (0.5 mg/kg, intraperitoneally) and performed the rotational test, where the 6-OHDA group showed worsening rotations compared to the safflower group (35 and 70 mg/kg/day, oral administration for three weeks) plus 6-OHDA and Madopar groups [121]. Giuliano et al. (2020) administered 20 µg 6-OHDA (dissolved in 3 µL at 1 µL/min injection rate) unilaterally into the right striatum in male Sprague Dawley rats to assess the lignan 7-hydroxymatairesinol (10 mg/kg daily with food for 28 days) effect on the motor impairments by applying the apomorphine-induced (0.5 mg/kg, intraperitoneally) rotational and cylinder tests. The lignan improved the behavioral impairments and slowed the progression of dopaminergic terminals’ degeneration in the striatum of 6-OHDA plus lignan 7-hydroxymatairesinol fed group [122]. Wattanathorn and Sutalangka (2015) administered 6 µg of 6-OHDA (in 0.2% ascorbic acid saline, 2 µL) into right substantia nigra in male Wistar rats, they revealed the efficacy of the combined extract of *Cyperus rotundus* and *Zingiber officinale* (at 100, 200, and 300 mg/kg daily oral administration for 14 days after 6-OHDA) against 6-OHDA neurotoxicity toxicity. Their data showed for the behavioral tests such as apomorphine-induced (0.5 mg/kg, subcutaneously) rotational test and Morris water maze test for spatial memory was improved motor performances in *C. rotundus* and *Z. officinale* group plus 6-OHDA group compared to 6-OHDA alone group [123]. Chen et al. (2017) administered 4 µg/2 µL 6-OHDA intrastriatal in male C57BL/6 mice and tested the effect of CXC195-tetramethylpyrazine derivative (daily for 7 or 14 days, intraperitoneally after 6-OHDA) against 6-OHDA-induced neurotoxicity. They observed increased apomorphine-induced (0.1 mg/kg, subcutaneously) rotational behavior in the 6-OHDA-induced group compared to normal control and other experimental groups, and the CXC195-tetramethylpyranzine derivative-treated mice ameliorated the behavioral deficits caused by 6-OHDA [124]. Ren et al. (2017) investigated the effects of FTY720 (2-amino-2-[2-(4-octylphenyl) ethyl] propane-1,3-diol) (0.5 mg/kg, intraperitoneally for seven days before the lesion) against 6-OHDA-induced neurotoxicity in male C57BL/6 mice. For in vivo studies, they administered 6 µg of 6-OHDA (in 2 µL of normal saline with 0.02% ascorbic acid) intrastriatal injection into the right striatum and assessed the pretreatment with FTY720 to 6-OHDA lesioned mice improved better both the motor abnormalities (apomorphine-induced rotation test: 0.1 mg/kg, subcutaneously), nigral dopaminergic damage, and associated inflammation with the activation of AKT and ERK1/2 pro-survival pathways [125]. The drug dose, lesion-type, and behavioral evaluating methods in each 6-OHDA-induced PD animal model are summarized in Table 2.

### 3.2. MPTP

**Figure 5:**
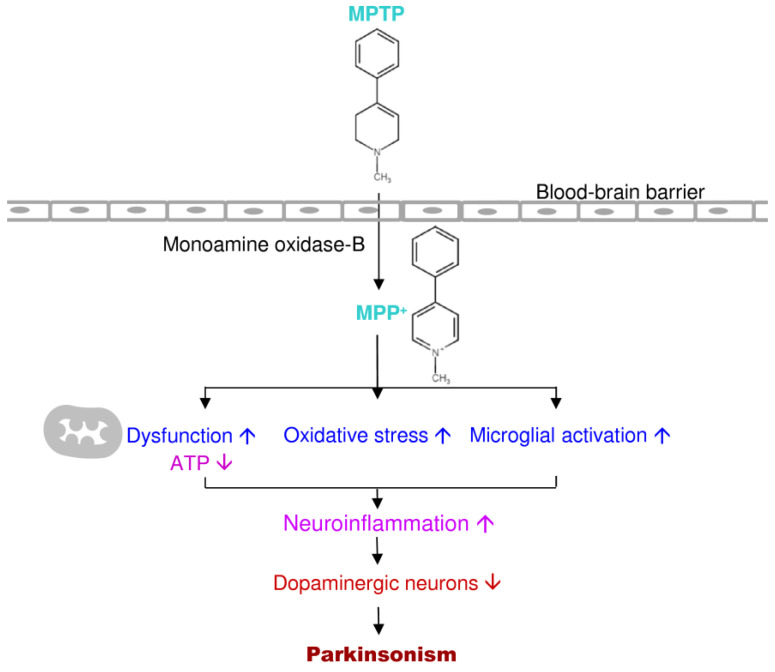
Putative mechanism of action of 1-Methyl-4-phenyl-1,2,3,6-tetrahydropyridine (MPTP) (and MPP^+^) in PD progression.

MPTP is a relatively simple compound that significantly impacts the understanding and treatment of PD over the past 30 years [126]. MPTP is a byproduct of a synthetic heroin 1-Methyl-4-phenyl-4-propionoxypiperidine. In 1982, seven youth individuals acquired severe PD after they used MPTP themselves [126,127]. Ballard et al. (1985) found MPTP in the synthetic heroin causes selective destruction of dopaminergic neurons of the nigrostriatal pathway to produce PD symptoms in humans and other primates [127,128]. MPTP is a lipophilic compound; it can pass the blood-brain barrier into the brain and be rapidly converted to the toxic metabolite, 1-Methyl-4-phenylpyridinium or MPP^+^ by monoamine oxidase-B [126]. Then MPP^+^ is selectively taken up by dopaminergic neurons through dopamine transporters and inhibits complex I of the mitochondrial electron transport chain, causes Parkinsonism [126,129]. MPP^+^ severely prompts the mitochondrial respiratory defects; it blocks ATP synthesis and activates free radical formation in mitochondria of dopaminergic neurons [130,131]. The MPTP-induced animal model and human PD showed similar conditions in the pathogenesis (Figure 5).

Uchida et al. (2015) administered 2.0 mg/kg of MPTP for five days, subcutaneously in common marmosets, and evaluated the anti-Parkinsonian property of istradefylline (10 mg/kg, oral administration) in combination with low levodopa dose (2.5 mg/kg, oral administration). They also tested the therapeutic efficacy of two dopamine agonists: ropinirole (0.025–0.075 mg/kg, oral administration) and pergolide (0.01 mg/kg, oral administration). They found that the adenosine A2A receptor antagonist istradefylline enhances anti-Parkinsonian activity induced by combined treatment with low doses of levodopa and two dopamine agonists in MPTP-treated common marmosets [132]. Their results indicated that the istradefylline addition in the treatment of PD benefits in controlling motor symptoms [132]. Lin et al. (2020) administered 10 mg/kg/day of MPTP intraperitoneally for three days in male C57BL6 mice and evaluated the celastrol effect against MPTP-induced neurotoxicity. The cylinder test on the 11th day and rotarod test on the baseline and 11th day revealed that the MPTP group reduced forelimb usage and latency to fall (increased on the 11th day) compared to normal control and celastrol-treated mice [13]. Celastrol (3 mg/kg/day for three days) enhanced better PD motor symptoms induced by MPTP compared to MPTP alone treated mice [13]. Lin et al. (2017) administered 10 mg/kg of MPTP intraperitoneally for three days in male C57BL6 mice and evaluated the electroacupuncture stimulation against MPTP-induced neurotoxicity. Rotarod test for behavior analysis before the electroacupuncture and the after MPTP administration (on the 8th day), electroacupuncture (50 hertz) stimulation at GB34 (Yanglingquan) and LR3 (Taichong) acupoints decreased the motor abnormality significantly in MPTP mice compared to MPTP alone treated mice [133]. Kim et al. (2019) administered 20 mg/kg of MPTP, intraperitoneally in male C57BL/6J mice and evaluated the purified bee venom phospholipase-A2 against MPTP-induced neurotoxicity. Bee venom phospholipase-A2 treatment defeated the motor deficits (pole test) and inhibited loss of dopaminergic neurons within the substantia nigra in a dose-dependent manner in PD mice [134]. Liu et al. (2020) administered MPTP at 15 mg/kg intraperitoneally to female mice and evaluated the effect of ACT001 (a fumarate salt of dimethylaminomicheliolide and a derivative of parthenolide) against Parkinsonian abnormality in female BALB/c mice. ACT001 (20 mg/kg, intragastric administration for seven days) in combination with levodopa (5 mg/kg) in MPTP lesioned mice, the behavioral tests for cylinder and open-field locomotion activity (on the 8th day) resulted that the better recovery from MPTP-induced motor abnormality and dopaminergic neurodegeneration compared to MPTP-induced group and, suggested that the ACT001 in combination with levodopa (5 mg/kg) which reduced the dose of levodopa in PD [135]. Liu et al. (2019) showed that the resveratrol (10 mg/kg, oral administration) in combination with levodopa (5 mg/kg, intraperitoneally) protect female BALB/c mice from MPTP-induced motor impairment, the behavioral tests for the open-field locomotion activity test (on the 8th day) and the rearing test (on the 9th day) showed inhibited the motor dysfunction compared to MPTP group. The MPTP mice showed the loss of dopaminergic neurons and weakened astroglial stimulation in the nigrostriatal pathway and demonstrated that the co-administration of resveratrol along with levodopa (5 mg/kg) were identical to the administration of 8 mg/kg levodopa in MPTP-induced PD mice, thereby lesser side effects [136]. Biju et al. (2018) administered 125 mg/kg of MPTP (25 mg/kg each dose at 3.5-day intervals for five weeks) subcutaneous injections in male C57BL/6J mice models and evaluated the methylene blue (1 mg/kg/day from 11th–100th day with drinking water) against MPTP-induced neurotoxicity. The methylene blue treatment ameliorated MPTP-induced motor dysfunction assessed for the open-field test, static bar test, horizontal bar test, olfactory discrimination, and nesting behavior test [137]. Datta et al. (2020) administered MPTP at the dose of 0.1 mg/nostril at a seven-day interval (Baseline, 7th and 14th day) in male albino Wistar rats and evaluated against MPTP-intranasal administration on liver functions and transition from non-motor to motor impairments. MPTP caused motor imbalance with a single dose; the repeated doses lead to worsening of motor balance in the rats and showed that the locomotor activity affected from the second MPTP dose increased the imbalance by the third dosage [138]. Roostalu et al. (2019) administered MPTP at 20 mg/kg, intraperitoneally in male C57Bl/6 mice. The rotarod test resulted in the imbalanced motor behavior in MPTP-group mice compared to the control group on the 6th day after MPTP administration. Decreased tyrosine hydroxylase-positive signals in the substantia nigra, caudate-putamen, globus pallidus, and subthalamic nucleus also observed the limbic regions (amygdala and hypothalamus) increased expressions of tyrosine hydroxylase signal intensity [139]. Chen et al. (2017) administered 15 mg/kg of MPTP for four times, intraperitoneally at 2-h intervals (60 mg total) in female C57BL/6 mice and evaluated the icariin (active constituent of *Epimedium sagittatum*) effect against PD. The motor impairments (rotarod test on the 9th day after MPTP injection) that MPTP significantly worsened the residence time, though the pretreatment with the icariin in 50, 100, and 200 mg/kg groups showed betterment in motor performance dose-dependently [140]. Zheng et al. (2017) injected 30 mg MPTP/kg/day, intraperitoneally for eight consecutive days in male mice, and evaluated the paeoniflorin (a bioactive compound from *Radix Paeonia Alba* root) effects in male C57BL/6 mice. Behaviorally impaired MPTP mice for motor activity and rotarod performances were restored by paeoniflorin (15 or 30 mg/kg, intragastric) and Madopar (100 mg/kg, intragastric) administration [141]. Ko et al. (2016) administered intravenous injections of 0.2 mg/kg of MPTP until the PD symptoms in captive-bred monkeys. The behavioral assessments (motor and cognitive task performances test) in levodopa-treated MPTP-induced macaque monkeys that revealed the benefits of istradefylline treatment in PD behavior deficits in a dose-dependent manner [142]. Overall, they concluded that treatment with istradefylline (75 or 100 mg/kg, oral administration) plus levodopa alleviated better with less dyskinesia, reduced the levodopa-induced cognitive dysfunction and its usage in PD [142].

Zhou et al. (2018) administered 30 mg of MPTP/kg doses once a day intraperitoneally, for five consecutive days to in male C57BL/6 mice models to evaluate the (−)-epigallocatechin-3-gallate effect against MPTP-induced neurotoxicity. They administered the (−)-epigallocatechin-3-gallate with water, one day before MPTP treatment to the 20th day after MPTP administration and applied the behavioral test (pole test) and observed the impaired motor behaviors in MPTP-induced mice models, where the impairments were restored in MPTP mice treated with (−)-epigallocatechin-3-gallate (50 mg/kg) [143]. Zheng et al. (2019) administered 30 mg MPTP/kg intraperitoneally for five consecutive days in male C57BL/6 mice models. They revealed the effect of baicalein with low dose levodopa (25 mg/kg, intraperitoneally) on MPTP-induced mice. For the behavioral tests, they applied gait analysis on the 21st day of the experimental period by the catwalk and observed gait deficits on dynamic paw function and posture stability in the MPTP group. The levodopa (25 and 50 mg/kg, intraperitoneally) restored the MPTP prompted gait discrepancies, and the effect showed positively dose-dependent [144]. Yue et al. (2016) administered a low dose of 0.2 mg MPTP/kg through the lower extremity vein until the onset of PD symptoms (i.e., on the 10th–13th day after MPTP-Kurlan score increased to ~10) in cynomolgus monkeys. Upon the onset of PD, they assessed the behavioral tests using the video recording, clinical rating, and measurement of overall home-cage activity levels and observed that the PD progression was at peak during the 3rd–12th day, and then the Kurlan score plateaued (>15). Subsequently to the rapid PD progression, a Kurlan score > 15 and at the end of the 3rd month showed a weakened risk of spontaneous rescue [145]. They observed stable PD symptoms for three months and suggested the model as an effective PD model for investigating the Parkinsonian [145]. Seo et al. (2019) administered 0.2 mg MPTP/kg intramuscularly in female *Macaca fascicularis* and observed MPTP toxicity through the decrease of global activity, dopamine transporter activity, and increased PD impairment scores from the 4th–48th week after the first MPTP injection. They showed global activity using a video-based analysis system with stable PD symptoms. Their experiments were correlated with global activity and Parkinsonian behavior scores, along with dopaminergic neuronal loss (by immunohistochemistry and western blot) in the basal ganglia [146]. Franke et al. (2016) administered a total of 2.5 mg of MPTP (0.5 mg/once a day/week) subcutaneously in marmosets. The behavioral stress increased during MPTP exposure and recovered after the completion of MPTP administration. The spontaneous locomotor activity also decreased in the bungalow test (from 23 ± 5–14 ± 3) during MPTP administration. The marmosets recovered during the last four weeks of the experimental recovery period and revealed no changes in the level of dopamine, serotonin, nor-adrenalin in the caudate nucleus, protein expressions in the brain after the recovery period [147]. Nielsen et al. (2016) administered 10 mg of MPTP/mL subcutaneously implanted (continuous infusion, delivery of 4–24 mg MPTP/day for 11 weeks) on the right side of the back in female Gottingen minipigs. MPTP caused abnormal behavior in all of the MPTP-induced animals and showed PD symptoms with impairments in the bradykinesia, rigidity, coordination, and chewing complications. They also observed the stable symptoms only in between the dose 12 and 18 mg MPTP/day groups. Their results for digital gait analysis, high-performance liquid chromatography, and stereological counts of tyrosine hydroxylase positive neurons in the substantia nigra showed a dose-dependent reduction in the gait velocity, metabolites level of the striatum, and the neuronal numbers with higher MPTP doses [148]. They also observed an increase in α-synuclein immunohistochemical staining with higher MPTP dosages and suggested that the Göttingen minipigs categorized to the largest-animal model of MPTP administration with unchanging Parkinsonian deficits (at 18 mg/day) [148]. Hwang et al. (2019) administered 10, 20, and 30 mg of MPTP/kg, intraperitoneally four times per day at one-hour intervals in male C57BL/6 N mice. MPTP administration resulted in impairments in motor performances (rotarod and pole test) and the nigrostriatal neurotoxic responses (dopamine, tyrosine hydroxylase positive, and protein carbonylation) [149]. They observed a similar neurotoxic PD response and locomotor abnormalities impaired dose-dependently [149]. Hu et al. (2020) administered 15 mg of MPTP/kg, intraperitoneally four times (a cumulative of 60 mg/kg) with or without lipopolysaccharide (5 mg/kg, intraperitoneally) in male C57BL/6 mice models and evaluated the Hua-Feng-Dan and *Rannasangpei* effects against MPTP neurotoxicity. Muscle coordination balance by rotarod and revealed that MPTP and lipopolysaccharide-treated mice showed severe motor impairment compared to MPTP or lipopolysaccharide alone-treated, whereas the Hua-Feng-Dan and *Rannasangpei*-treated mice restored the impairments caused by MPTP-induction [150]. Yun et al. (2015) administered 7 mg/kg of MPTP subcutaneously in male marmosets and evaluated the adverse effects of MPTP. The tower test (natural behavior, jumping, and captive height preference) revealed that the severely impaired marmoset’s in the jumping ability not reached high levels in the tower test compared to their baseline values [151]. They observed an average level reached 1–3 in 7 min, and the loss of jumping ability was not recovered in 32 weeks in MPTP-induced marmosets [151].

Arbez et al. (2020) administered a cumulative of 5 mg/kg MPTP (2.5 mg/kg, two separate injections) subcutaneously in male mice (*G2019S* transgenic mice) and evaluated the *G2019S*-LRRK2 mutation against MPTP toxicity. They revealed that the homozygous mice did not show motor abnormalities, whereas the reduced open-field activity in the transgenic mice at six months of age, and the defect increased at 12 months. Besides, the rotarod test results exhibited a significant decrease at 9 and 12 months of age. The motor deficits of transgenic mice (*G2019S*-LRRK2) showed to be advanced and age-dependent since the mice did not show any alterations at four months’ age. The wild-type LRRK2 transgenic mice showed the motor abnormalities even at 12 months of age but significantly differed compared to control mice. Their data showed that the LRRK2 overexpression in mice escalated dopaminergic neurons’ loss when exposed to MPTP doses and suggested that the *LRRK2* gene/MPTP interaction mice model could be a useful tool for better understanding in PD investigations [152]. Guo et al. (2016) administered a cumulative dose of 80 mg/kg MPTP (20 mg/kg, four intraperitoneally) in male C57BL/6 mice models. They applied locomotor activity and rotarod tests showed that the stemazole high dose, Madopar (120 mg/kg/day, seven days, intragastric), stemazole low or medium-dose groups mice improved their locomotor activity compared to MPTP-lesioned mice [153]. Rinaldi et al. (2019) administered a cumulative 100 mg of MPTP/kg intraperitoneally in male C57Bl/6 J mice models and evaluated the inPentasomes effect against MPTP. They applied the open-field test and pole test to analyze the motor deficits in mice and revealed that the inPentasomes intranasal administration (1 or 4 μg/kg) inhibited the motor impairments by MPTP in a dose-dependent manner [154]. Nataraj et al. (2016) administered a cumulative dose of 120 mg of MPTP/kg, intraperitoneally (30 mg/kg, four injections) in male C57BL/6 mice, and evaluated the Lutein protective effect against MPTP. They assessed the behavioral analysis using the open-field test, narrow beam test, hang test, and catalepsy tests. The behavioral tests revealed that MPTP administration prompted severe motor impairments (hind-limb coordination and memory dysfunction) due to the loss of striatal dopamine [155]. Zhou et al. (2019) described the noninvasive ultrasound deep brain stimulation of subthalamic nucleus/globus pallidus improved motor functions in MPTP-induced male C57BL/6 J mice. Subthalamic nucleus/globus pallidus-ultrasound deep brain stimulation increased the latency to fall in the rotarod test on the 9th day and reduced the pole test time on the 12th day. Their data also showed that the ultrasound deep brain stimulation protected the dopaminergic neurons by reducing antiapoptotic expression [156]. The drug dose, lesion type, and behavioral evaluating methods in each MPTP-induced PD animal model are summarized in Table 3.

### 3.3. MPP^+^

As previously described, MPP^+^ is a neurotoxic cation metabolite from MPTP used to induce PD in experimental models [157]. In addition to being taken up by the plasma membrane dopamine transporter, MPP^+^ also undergoes high-affinity uptake by the vesicular monoamine transporter-2 [158]. Intrabrain injection of MPP^+^ induces dopaminergic neuronal cell death in the brain by inhibiting NAD(H)-related oxidation of mitochondrial complex I and electron transport chain [133]. After MPP^+^ impairs the mitochondrial function, MPP^+^ affects the processes of dopamine release and subsequent formation of hydroxyl radicals [159,160].

Moretti et al. (2015) administered 1.8 µg of MPP^+^ per site intracerebroventricularly in male C57BL6 mice and evaluated the effect of agmatine (oral administration) against MPP^+^-induced neurotoxicity. They applied the behavioral tests using tail suspension test, open-field test, and splash test, where the data revealed that the agmatine protected the progression of depressive and impaired motor behaviors induced by MPP^+^ toxicity in mice [161]. Cunha et al. (2017) revealed that MPP^+^ (1.8–18 µg/mouse, intracerebroventricular) dose-dependently induces behavioral discrepancies in male C57BL6 mice; they observed that high dose MPP^+^ group reduced the number of rotations in open-field test and the time of latency to fall in rotarod test and MPP^+^ at a low dose (1.8 µg/mouse) did not alter the locomotion [162]. Lin et al. (2017) administered 8 µg/rat of MPP^+^ unilaterally in Sprague Dawley rats and evaluated the effect of electroacupuncture against MPP^+^-induced neurotoxicity. They applied apomorphine-induced (5 mg/kg, intraperitoneally) rotational and locomotor behavior after the 8th day of MPP^+^ injection, found that electroacupuncture (50 hertz) stimulation at GB34 (Yanglingquan) and LR3 (Taichong) acupoints decreased the motor abnormality in MPP^+^ rats compared to MPP^+^ alone-treated rats [133]. Pérez-Barrón et al. (2015) administered 10 µg of MPP^+^ (in 8 µL saline) intrastriatal into the right striatum in male Wistar rats and evaluated the efficacy of *Buddleja cordata* methanolic extract (50 or 100 mg/kg, oral administration, pretreatment for 14 days). They applied the apomorphine-induced (1 mg/kg, subcutaneously) circling behavior test and observed severe behavioral impairments in MPP^+^-induced rats, whereas the impairments were minimized in *B. cordata* methanolic extract pretreated rats in a dose-dependent manner [163]. Rubio-Osornio et al. (2015) administered 10 µg of MPP^+^ (in 8 µL saline) intrastriatal into the right striatum to induce the neurotoxicity in male Wistar rats and assessed the epicatechin activity (40, 60, and 80 mg/kg, oral administration for five days). They applied an apomorphine-induced (1 mg/kg, subcutaneously for six days after MPP^+^-injection) circling behavior test and revealed that MPP^+^-induction increased the turning behaviors, where the subchronic dose of epicatechin at 100 mg/kg decreased the turning behavior in MPP^+^-induced rats [164]. Aguirre-Vidal et al. (2017) administered 15 µg of MPP^+^ (in 8 µL saline) intrastriatal on the 6th day, into the right striatum in male Wistar rats and evaluated the effect of β-estradiol-3-benzoate (100 µg/kg, for every 48 h for 11 days) against MPP^+^ toxicity. They applied apomorphine-induced (1 mg/kg, subcutaneously for six days after MPP^+^-injection) circling behavior test and showed that MPP^+^-induced rats circling behavior became higher compared to normal control rats, whereas the β-estradiol-3-benzoate-treated rats (gonadectomized 30 days’ prior) reduced the circling behavior [165]. The β-estradiol-3-benzoate-treated rats also inhibited the loss of dopamine, reduced the lipid peroxidation, increased the tyrosine hydroxylase-positive in the substantia nigra of MPP^+^-lesioned rats [165]. Aguirre-Vidal et al. (2015) administered 15 µg of MPP^+^ (in 8 µL saline) intrastriatal on the 6th day, into the right striatum in male Wistar rats and evaluated the effect of lovastatin (5 mg/kg, intraperitoneally, seven days). They applied an apomorphine-induced (1 mg/kg, subcutaneously six days after MPP^+^ injection) circling behavior test and revealed that MPP^+^-induced rats circling behavior became higher than normal control rats, whereas the lovastatin-treated rats reduced the circling behavior [166]. Chen et al. (2017) administered 30 µg of MPP^+^ (in 4 µL saline) unilaterally into the striatum in Sprague Dawley rats and evaluated the transforming growth factor-β1 (10 ng or 50 ng, intracerebroventricular after 14 days of MPP^+^). They applied apomorphine-induced (1 mg/kg, six days after MPP^+^-injection) rotational test, open-field test, and rotarod test, which showed impairments induced by MPP^+^, where the transforming growth factor-β1 (50 ng) administration rescued motor balances in a dose-dependent manner [167]. The drug dose, lesion type, and behavioral evaluating methods in each MPP^+^-induced PD animal model are summarized in Table 4.

### 3.4. Paraquat

**Figure 6:**
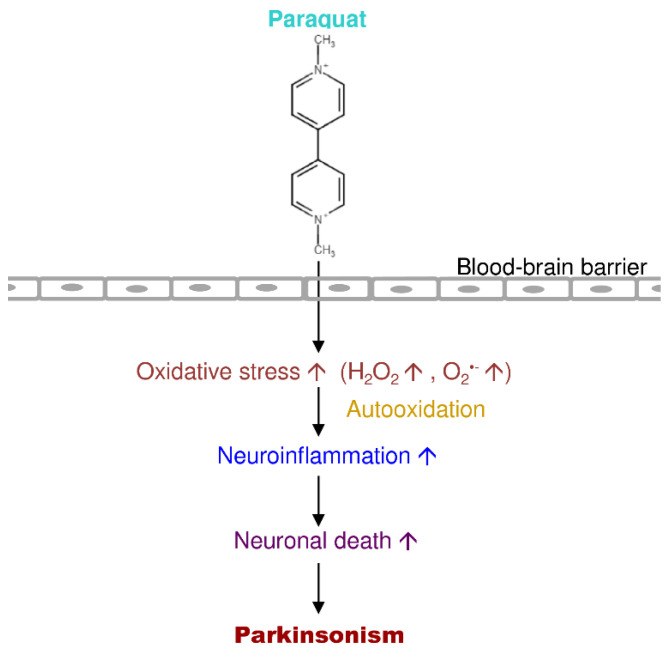
Putative mechanism of action of paraquat in PD progression.

Paraquat is an extremely toxic quaternary nitrogen herbicide [168]. Several studies demonstrated that paraquat offers severe damage to the lung, followed by a reparative phase dominated by extensive fibrosis [169]. Paraquat is a widely used pesticide due to its low cost and rapid action [170]. Numerous cases of acute paraquat intoxication and death were accounted for over the past few decades. Paraquat accumulates in the lung and the kidney, where it uses its significant acute toxicological effects after the absorption, free from the route of exposure [171]. Moreover, paraquat is very poorly absorbed and is excreted nearly unchanged in the urine [172]. Previous reports showed that the metabolism of paraquat occurs through demethylation or oxidation (pyridone and dipyridone ion) (Figure 6) [173,174,175].

Philippot et al. (2019) administered 0.2 or 0.02 mg of paraquat per kg during postnatal 10th and 11th day; besides, they were also exposed to 100 or 300 milligray gamma radiation for two-hours to achieve the neurotoxic model in male C57Bl/6 mice. They applied the behavioral tests such as spontaneous behavior test, radial-arm maze test and revealed that the severe impairments showed in mice irradiated with 300 milligray and paraquat than normal control mice [176]. Their data on gamma irradiation (300 milligray) combined with paraquat produced a potent stimulation of neurological defects in mice [176]. Rudyk et al. (2017) administered paraquat at 1 mg/kg or 10 mg/kg, intraperitoneally on the 1st, 5th, 8th, 12th, 15th, and 19th day in male C57Bl/6 mice. They observed the high dose paraquat group mice showed impairment in the behavioral functions (home cage locomotor activity, sucrose preference test, spontaneous alternation behavior Y-maze test, open-field test, elevated plus maze test, and forced swim test) than the low dose group (control mice: no impairments). They revealed that paraquat is a systemic stressor of corticoid signaling and motor balance impairments in mice [177]. Anselmi et al. (2018) administered 1 mg paraquat/kg (daily for seven days) orally in male Sprague Dawley rats. They also administered 0.05% of lectin combined with paraquat for seven days and observed PD symptoms and gastric dysmotility. They applied the vibrissae test and stepping test and found severe impairments in the paraquat plus lectin-treated group, whereas the levodopa (4 mg/kg) pretreated group improved in motor performance [178]. Rudyk et al. (2019) administered a cumulative of 60 mg paraquat/kg (10 mg/kg, twice a week for three weeks) in male C57Bl/6 mice models and assessed home cage locomotor activity, sucrose preference test, spontaneous alteration behavior Y-maze test, elevated plus maze test, rotarod test, and forced swim test. The rotarod test and increased signs of anhedonia (sucrose preference test), where paraquat-treatment induced some non-motor symptoms in open-field, Y-maze, and plus-maze test defects without significant signs between the group of mice. They suggested that there is no interaction (additive or synergistic) between chronic unpredictable stress exposure and paraquat on behavioral and non-motor behavioral effects (except the rotarod test and 5th week of sucrose preference) [179]. Cristóvão et al. (2020) administered paraquat 2.5 mg/kg/day, subcutaneously (osmotic minipump implanted in the back with 0.25 µL/h fluid delivery rate) for four weeks in male Wistar rats. They applied rotarod and open-field behavioral tests for analyzing motor impairments caused by paraquat [180]. Their results supported that paraquat-induced PD models showed an increased dopaminergic loss, neuroinflammation, oxidative stress, and α-synuclein pathogenesis, along with motor defects [180]. Ait-Bali et al. (2016) administered 20 mg/kg paraquat (in gramoxone 200 g/L) orally in male and female Swiss mice. Prenatal paraquat sub-lethal exposure disturbs the fertility and reproductive parameters in pregnant mice [181]. Their data showed the disturbances in the motor activity were due to a reduction in tyrosine hydroxylase positive neurons in the substantia nigra; while the rise of an anxiety level and weakened cognitive function paralleled the escalation in the glial fibrillary acidic protein–immunoreactive cells density in the hippocampus of the brain [181]. Heydari et al. (2019) administered 27 and 54 mg/m^3^ of paraquat eight times on the 1st, 3rd, 5th, 7th, 9th, 13th, and 15th day 30 min each aerosol exposure in Sprague Dawley rats and evaluated the effect of *Zataria multiflora*. Inhaled rats tested for the Morris water maze and passive avoidance, revealed the learning and memory were damaged in paraquat-inhaled rats. They treated the rats with *Z. multiflora* hydroalcoholic extract (two doses, 200 and 800 mg/kg/day gavaged, 16 days) and found it improved the behavioral, learning, memory, and lung impairments induced by paraquat [182]. Gonçalves et al. (2018) administered 5 mg/kg of paraquat intraperitoneally on the 2nd, 4th, 6th, and 7th day in Swiss male mice. Their data for open-field, rotarod, neurological severity score, and social recognition tests revealed that the systemic administration of lipopolysaccharide (3, 5, and 7 mg/kg, intraperitoneally) induced slower social recognition behavioral deficit and striatal stress in adult mice [183]. Chinta et al. (2018) administered a cumulative of 42 mg/kg of paraquat intraperitoneally (7 mg/kg at two-day intervals for a total of six doses) in C57Bl6/J (p16-3MR) mice models. They applied the cylinder test, the impaired rearing behavior observed in the paraquat group, whereas the cotreated group with ganciclovir (25 mg/kg, intraperitoneally for five days) rescued from the impairment caused by paraquat. Their results revealed that paraquat induces astrocytic senescence and a senescence-associated secretory phenotype both in vitro and in vivo [184]. They also suggested that exposure to environmental toxin paraquat promotes aggregation of substantia nigra cells’ senescence, which causes neurodegeneration in the brain [184]. The drug dose, lesion type, and behavioral evaluating methods in each paraquat-induced PD animal model are summarized in Table 5.

### 3.5. Rotenone

**Figure 7:**
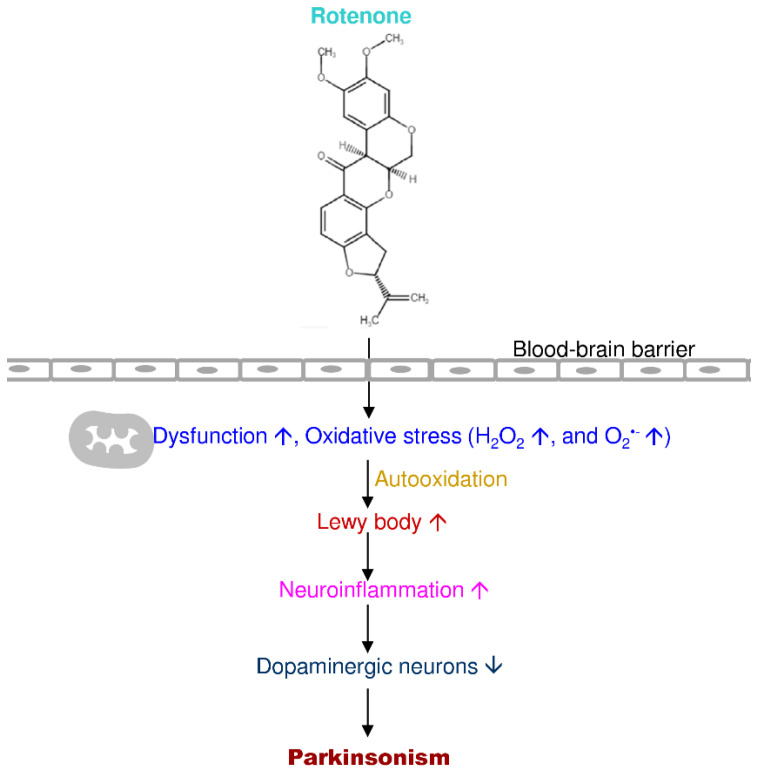
Putative mechanism of action of rotenone in PD progression.

Rotenone is a naturally occurring organic insecticide that can damage the mitochondrial complex I electron transport chain to induce mitochondrial dysfunction and lead to dopaminergic neuronal loss in the nigrostriatal pathway and reactive oxygen species accumulation [185,186]. Rotenone toxicity depends on the mitochondrial complex I interaction, but rotenone-insensitive cells such as NDI1 (single-subunit NADH dehydrogenase of yeast) are unaffected by the toxicity of rotenone [186]. Thus, electrons from mitochondrial complex I are shifted via NDI1 to the downstream electron transport chain for mitochondrial respiration [187]. Rotenone-induced PD models imitate experimental features of idiopathic PD and run through the slow and progressive damage of dopaminergic neurons and the Lewy body establishment in the nigral-striatum [188]. There are various exposure routes such as oral administration, subcutaneous injection, osmotic pumps, intraperitoneal injection, and intravenous administration—many studies used administration subcutaneously for obtaining PD models in rats. The autopsy studies showed more robust evidence for the association of hyperoxidation and protein carbonyl production in rotenone-induced PD pathogenesis (Figure 7) [189].

Yu et al. (2017) administered 0.5 mg rotenone/mL, intraperitoneally for 35 days to induce the neurotoxicity in Sprague Dawley rat pups. They tested the co-administration of iron (120 µg/g, intraperitoneally) with rotenone consecutively for 35 days and tested behavioral parameters at 14 weeks. Their cotreated iron plus rotenone group showed worse neurochemical and motor deficits in male and female rats [190]. Zhang et al. (2017) administered rotenone at the dose of 1.5, 2, or 2.5 mg/kg/day for five weeks (in sunflower oil) in male Wistar rats. They applied rearing, catalepsy, rotarod, and locomotor tests and found the rotenone-induced group showed lesser rear activity than vehicle and control groups. They also showed that the subcutaneous injections of rotenone at 2 mg/kg/day affected the behavioral features and molecular mechanisms of substantia nigra with Lewy body aggregation in rats [191]. Bandookwala et al. (2019) administered 2 mg/kg of rotenone subcutaneously in male Sprague Dawley rats. The rotenone-induced group’s grip strength showed decreased muscle strength compared to control rats on the 20th day after rotenone administration. The combined treatment of edaravone (1.5 mg/kg, intraperitoneally) and caffeine (10 mg/kg, intraperitoneally) was rescued against grip strength in the rotenone-induced group over 20 days. In the Y-maze test, rotenone produced spatial memory loss in rats on the 20th day, whereas the edaravone–caffeine-treated group represented a slight recovery of spatial memory caused by rotenone [192]. Overall, the study revealed that edaravone–caffeine was rescued from motor impairments and prevented the oxidative damage in rotenone-treated rats [192]. Palle and Neerati, (2018) administered rotenone at the dose of 2 mg/kg rotenone, subcutaneously for 35 days in male albino Wistar rats, and evaluated the effect of resveratrol nanoparticles. They used rearing and rotarod test to analyze motor impairments; they found rotenone worsen impairments in rearing and rotarod activity than resveratrol (40 mg/kg, oral administration, 30 min before rotenone injection for 35 days) plus rotenone-induced group. The resveratrol nanoparticle group (40 mg/kg, oral administration, 30 min before rotenone injection for 35 days) showed better recovery in motor activities against rotenone-induced rats. They revealed that the resveratrol nanoparticle treatment benefits against rotenone toxicity by inhibiting motor deficits, preventing oxidative damage, modulating tricarboxylic acid cycle enzymes, and mitochondrial complex I activity [193]. Alikatte et al. (2020) administered rotenone at the dose of 2 mg/kg/day, subcutaneously for 35 days in male albino Wistar rats, and evaluated the effect of fisetin (oral administration 10 or 20 mg/kg/day for 25 days). They applied a cylinder test on the 36th day and revealed that rotenone caused severe behavior impairment, whereas the rats treated with fisetin showed minimal (dose-dependent manner) motor dysfunctions in rats [194]. Darbinyan et al. (2017) administered rotenone at the dose of 2.5 mg/kg, intraperitoneally for 21 days in male albino Wistar rats, and evaluated the effect of curcumin (200 mg/kg/day, intraperitoneally for 21 days). They applied the cylinder behavioral test to assess the behavioral impairments and revealed that rotenone administration impaired motor functions, whereas the curcumin treatment restored the behavioral dysfunction significantly from the 3rd week of treatment in rats [195]. Dhanalakshmi et al. (2016) administered rotenone at the dose of 2.5 mg/kg/day, intraperitoneally for 10 days in male albino Wistar rats, and assessed the vanillin (oral administration) effect. They applied open-field, sucrose preference, and forced swim tests; they revealed that rotenone caused severe impairments in motor behavior and non-motor symptoms. Rotenone induction also reduced sugar intake, slower swimming activity, and increased latency behavior in rats [196]. Badawi et al. (2017) administered rotenone at the dose of 3 mg/kg/day, subcutaneously for 10 days in male albino rats, and evaluated the effect of sitagliptin and liraglutide. They applied cylinder test and catalepsy test; they revealed the progressive behavioral deficits caused by rotenone. The motor deficits caused by rotenone were minimized significantly by sitagliptin (30 mg/kg/day, oral administration) and liraglutide (50 µg/kg, subcutaneously) treatments in rats [197]. Carriere et al. (2016) administered rotenone at the dose of 4.0 µg/site for three sites (total 12 µg rotenone) unilaterally into the right striatum in male Sprague Dawley rats. They assessed the neuropathological effects of intrastriatal infusion of rotenone (as a rat PD model), which did not influence rats’ well-being over the experimental study. Rotenone produced motor impairments, whereas the chronic melatonin (4 µg/mL with drinking water, one week before the lesion and up to the 9th week till the sacrifice) administration ameliorated the apomorphine-induced (0.25 mg/kg, subcutaneously) behavioral akinesia and reversed the degeneration of the nigrostriatal pathway [198]. The drug dose, lesion type, and behavioral evaluating methods in each rotenone-induced PD animal model are summarized in Table 6.

### 3.6. Permethrin

**Figure 8:**
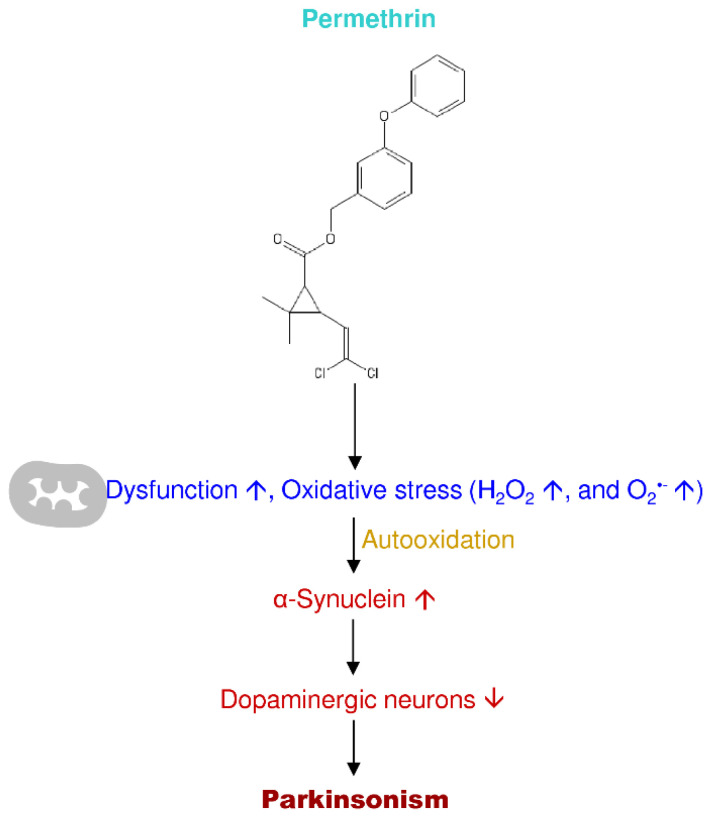
Putative mechanism of action of permethrin in PD progression.

Permethrin is widely used in household and agricultural repellents worldwide [199]. Chemically, it is a pyrethroid insecticide, contains a dichrolovinyl group and phenonthrin as isobutenyl group in the acid moiety that is more effective in humans if exposed [200]. Exposure to permethrin at earlier ages mainly affects the central nervous system, leading to progressive time-dependent damage of striatal dopaminergic neurons, mitochondrial dysfunction, and motor impairment directing Parkinsonism in old age [201]. Permethrin induces neurotoxicity through various mechanisms such as inflammation, oxidative stress, mitochondrial dysfunction (complex I), and cell death [202,203,204] (Figure 8).

Saito et al. (2019) exposed C57BL/6 mice to permethrin (0.3 ppm) in drinking water all through the prenatal and postnatal periods and studied the effects on the central nervous system in adult male mice [205]. They applied open-field test, light/dark transition test, and contextual/cued fear conditioning test for behavioral analysis and found that early life exposure to low levels of permethrin caused motor, learning, and memory impairments in mice [205]. Nasuti et al. (2017) administered permethrin at the dose of 34 mg/kg daily from the postnatal 6th–21st days and evaluated the effect of early exposure of permethrin-induced PD in Wistar rats. They applied the rotarod, footprint and beam walk, and T-maze tests (from postnatal 50th, 100th, and 150th day) and found early life exposure to permethrin impaired motor functions at adolescent age [206]. The drug dose, lesion type, and behavioral evaluating methods in each permethrin-induced PD animal model are summarized in Table 7.

## 4. Neurotoxin-Induced Experimental In Vivo Models of PD

Neurodegenerative diseases can be modeled in animals using consistent procedures that result in specific pathogenic events and behavioral outcomes [207]. The improvement of PD animal models is essential for assessing novel neuroprotective agents and therapeutic strategies. PD animal models mimic Parkinson-like pathology characteristics and imitate specific features of the neurological disease [208]. In these features, Lewy bodies in dopaminergic neurons are the main characteristic of PD pathogenesis [209]. Moreover, excessive striatal dopamine insufficiency causes easily noticeable motor retardation, bradykinesia, rigidity, and resting tremor, which are the foremost symptoms of PD [210]. A wide variety of animal models have been used for many years to analyze or assess PD characteristics.

In PD investigations, rodents and non-human primates are chief resources. The restrictions of the models account while the interpretation of results [211]. Non-human primate PD models are functionally, physiologically, and behaviorally comparable to human PD [212]. These models are infrequently used due to cost and ethical concerns. However, rats and mice are frequently used in PD investigations [75]. Nevertheless, no neurotoxins or genomic models can entirely imitate pathology compared to humans’ pathophysiology [213]. Subsequently, ecological issues and hereditary liability are supposed to play a vital role in the commencement and progression of PD; henceforth, the most excellent favorable models may be that associate genetic model through toxins exposure.

The remaining challenge is still understanding why changes in various proteins with altered or uncertain physiological functions congregate to comparable pathologic phenotypes, which are also observed in idiopathic PD [214]. Conversely, familial, environmental, and idiopathic PD forms present some differences from both the histopathological and clinical perspectives [215]. For example, PD patients carrying *PARKIN*, *PINK1,* or *LRRK2* mutation does not always present Lewy bodies [216,217]. Moreover, patients differ in the age of onset, ailment severity, neurodegeneration progression, and symptoms (including motor and non-motor) [218,219].

Both 6-OHDA and MPTP are neurotoxic compounds that are the substrates for the dopamine transporter [22]. The 6-OHDA can also act as a substrate for the norepinephrine transporter; when it is used alone, 6-OHDA is not exclusively explicit for dopamine neurons [220,221]. Because of the larger size (easier for brain surgery and microinjection), richer behavior patterns, and greater translation correlation, 6-OHDA and MPTP (unilateral intracerebral microinjection or intranasal administration) are commonly used in rats rather than mice [78,211,222,223]. The rats also show rich behavioral outlines and an extraordinary relevance in translation. Many studies in PD animal models are attentive to the motor behaviors related to dopamine reduction. However, the clear molecular basis and the molecular pathways of PD cell death remain mysterious.

## 5. Discussion

Mitochondrial dysfunction-induced dopaminergic neuronal death in the substantia nigra causes decreased voluntary movements in PD [18]. During PD development, α-synuclein accumulation in Lewy bodies of dopaminergic neurons has become more common in the brain [17,18]. Recent literature (of the selected forty-one articles) illustrated 6-OHDA is a frequently used neurotoxin than other drugs for inducing PD-like symptoms. The 6-OHDA toxicity is mediated by dopamine aggregation and oxidative stress, which leads to quinone, indole derivatives, free radicals, and hydrogen peroxide production [224]. The quinone derivatives (catecholamines) bind covalently to nucleophilic groups of proteins capable of forming covalent cross-links between the proteins [225]. Moreover, free radicals and hydrogen peroxide formed leads to dopaminergic cell death in the brain [226,227].

In 6-OHDA-induced investigations, most of the literature showed behavioral tests such as rotational test, open-field test, elevated plus maze test, cylinder test, grid test, bar catalepsy test, forced swim test, sucrose preference test, abnormal involuntary movements test, olfactory discrimination test, etc. Rotation test is used to detect the severity of the nigrostriatal lesion animal models [86]. Many 6-OHDA models studies applied apomorphine (0.1–1 mg/kg, subcutaneously or intraperitoneally) as a motor impairment activator before the behavioral tests. The levels of dopamine in the striatum and substantia nigra correlate with apomorphine-induced rotational behavior [228]. Few studies demonstrated the amphetamine-induced (psychostimulant) (0.1–5 mg/kg, subcutaneously or intraperitoneally) rotational tests for Parkinsonism investigations. Methamphetamine is derived from its parental drug amphetamine, which is an indirect agonist of dopamine; it causes similar side effects as amphetamine [229]. Upon the amphetamine or methamphetamine administration produce excessive dopamine in the synaptic clefts, and this release triggers reverse dopamine transporter, which affects the severe changes in behavior [229]. The unilateral striatal lesion of 6-OHDA and the apomorphine-induced rotations are the best behavioral rotations to assess the dopamine depletion in the striatum and substantia nigra when compared to amphetamine-induced rotations [230]. However, 6-OHDA cannot penetrate the blood-brain barrier and lacks its effect on Lewy body aggregation, which is not a similar feature of human PD [231,232,233].

MPTP causes selective damage to the nigrostriatal pathway’s dopaminergic neurons after the injection in animal models [234]. The MPTP doses selected from twenty-five research publications published in the last five years showed 0.06 µg/mL to 30 mg/kg based on the lesion type of MPTP. MPTP lesioning types include subcutaneous, intraperitoneal, intramuscular, intranigral, intranasal administration, and intravenous injections. The minimum concentration of lesion type of MPTP by 0.06 µg/mL intranigral injection and the higher doses by 30 mg/kg intraperitoneally. MPTP lesioning mainly affects dopaminergic production by generating damage to mitochondrial complex I of mitochondria [235]. Many types of motor function tests assessed in MPTP-induced animals, such as rating scale, locomotor activity, pole test, cylinder test, open-field test, rearing test, static/horizontal bar test, olfactory discrimination, nesting behavior, rotarod test, grip strength, gait analysis, video recording for activity, tower test, oculomotor task, operant task, hang test, and catalepsy tests. MPP^+^ transformed from MPTP by the enzyme monoamine oxidase-B and acts as a chief toxic substance to dopaminergic cells, which causes the neurodegeneration of substantia nigra in the brain [126,234]. MPP^+^ doses from the last five years selected eight research publications showed 1.8–30 µg based on the different lesioning methods such as intracerebroventricular and intrastriatal administrations. The motor performances in MPP^+^-induced experiments showed the circling behavior, open-field test, apomorphine-induced rotational behavior, and rotarod test. However, MPTP toxicity decreases dopamine content and tyrosine hydroxylase in the brain but lacks the effect on Lewy body formation, which is not biochemically similar to PD in humans [236].

Herbicides (paraquat and rotenone) and pesticides (permethrin) are also used to induce neurotoxic degeneration in the brain. Paraquat is a quaternary nitrogen herbicide, which causes brain neurodegeneration and extensive fibrosis in the lungs, but lacks striatal dopamine denervation in some models [237,238,239]. Upon rotenone administration, it damages mitochondrial function (complex I inhibition), dopaminergic neurons, and nigrostriatal pathway [186]. Rotenone toxicity induces mitochondrial dysfunction in substantia nigra and the whole brain [186]. Paraquat doses from the last five years selected nine research publications showed 0.2–20 mg/kg based on lesion types (oral administration, intragastric, intraperitoneally, subcutaneous, etc.). The lesion type of aerosol administration ranged from 27 and 54 mg/m^3^ for eight times to induce the brain degeneration in Sprague Dawley rats (Table 5). In contrast, the rotenone doses showed 4 µg/site–3 mg/kg from the recent nine research publications, with different lesion types such as intraperitoneal, subcutaneous, and unilateral (intranigral or intrastriatal) injections (Table 6). As per Table 5 and Table 6, recent literature showed the behavioral tests in paraquat or rotenone-induced models are akinesia, postural instability, open-field, spontaneous behavior, radial-arm maze, home cage locomotor, sucrose preference, Morris maze, Y-maze, elevated plus maze, forced swim, vibrissae, stepping, tremor, negative geotaxis, surface right reflex, cliff avoidance, cylinder tests, etc. Permethrin mainly affects the central nervous system and causes progressive aggregation of α-synuclein, loss of dopaminergic neurons, and muscle spasms [240,241]. Permethrin doses from two publications that were published last five years showed 0.05–34 mg/kg/day by oral administration [205,206]. Early-life exposure to permethrin as progressive PD animal models produces PD symptoms such as loss of striatal dopaminergic neurons, mitochondrial dysfunction, and motor impairments in adult life stages. The behavioral tests applied in this type of PD model are rotarod, footprint, beam walk, light/dark transition, and fear conditioning tests [205,206].

These neurotoxins induce neurodegeneration in substantia nigra in animal models. Apomorphine and amphetamine are dopamine agonists with a different mode of action. Apomorphine stimulates dopaminergic receptors; amphetamine increases the release of dopamine and blocks transmitters [242]. Apomorphine and amphetamine-induced rotations should be consistent and can specify the lesion criteria that essential to be met. Amphetamine is useful for partial lesion depletions and lacks the specificity compared to apomorphine [86]. Amphetamine doses also required high up to 5 mg/kg, still no specificity. Due to this reason, apomorphine is most widely used in PD experiments to induce the rotations before the behavioral analysis in animal models, and apomorphine is accurate and reliable for the detection of specific loss of nigrostriatal dopamine. The doses of apomorphine indicated in Table 2 show 0.05–3 mg/kg, subcutaneously/intraperitoneally administered prior (approximately 30 min before) performing the rotational or behavioral tests in animal models. However, apomorphine is a remarkable drug that is extensively used for stimulating motor functions in PD. Non-pharmacological treatments like deep brain stimulation and acupuncture have been used to improve motor function in PD patients [133,156]. In accordance with the above-cited literature, the midforebrain lesion type is more effective in causing neurodegeneration and the symptoms compared to other administrative methods in animal models of PD. The main drawback of neurotoxins and animal models is that they differ from PD’s natural progression in humans.

Nearly all the clinical research trials are used to assess novel findings for the PD, and the results based on a single aspect, but the medications are prescribed in a combined of pharmacological, surgical, rehabilitation-based aspects to treat or manage PD patients. Of the 23,000 original research articles on PD from the year 1990–2018, 48% were in rats, 37% in mice, 10% in non-human primates, and 5% in non-mammalian models applied in investigations related to Parkinsonism [222]. These portions of data signify the basis for the pharmacological, neurosurgical, and rehabilitative methodologies to investigate the PD.

In the past few decades, non-mammalian models such as *Drosophila* and *Caenorhabditis elegans* used in a lesser percentage in the number of PD experimental studies [222]. Moreover, these models have both advantages and disadvantages. These models’ advantages are the easier gene manipulation, shorter reproductive cycle, cheaper for maintenance, and clear pathology. These animal models easily exhibit behavioral symptoms similar to PD’s human symptoms. However, the *Drosophila* and *C. elegans* are smaller in size, which lacks the comfortability in assessing the several motor functions, α-synuclein expression, and different connectivity of dopaminergic neurons, unlike in humans [243,244].

## 6. Future Perspectives

Many studies suggested that α-synuclein aggregation, abnormal protein clearance, mitochondrial dysfunction, and neuroinflammation play fundamental roles in disease onset and PD pathogenesis progression. Neurotoxins mainly affect dopaminergic neurons by dopamine transporters by inhibiting the mitochondrial complex I activity, and further cause neurotoxicity in the brain. In PD investigations, dosages, lesion types, neurotoxins used to induce PD, and the timing for behavioral studies are all critical factors for evaluating the treatment/management efficacy of drugs. Indeed, no single animal model for PD produced to date mimics all the hallmarks of Parkinsonism. Toxic animal models and transgenic animal models are available in PD research, but they also have disadvantages. Some transgenic mice lack nigrostriatal degeneration. The handling is also expensive. However, the consistent and reliable neurodegenerative animal models in the nigrostriatal pathway stay a significant shortage in PD investigations. Future studies focusing on the low neurotoxic dose models which mimic all Parkinsonian symptoms that resemble exactly as human PD are needed to be discovered for better investigations to identify novel therapeutic targets or treatment.

## Figures and Tables

**Table 1:** Neurotoxins, lesion types, pathogenesis, and symptoms of Parkinson’s disease (PD).

Neurotoxin	IUPAC Name	Lesion Types	Pathogenesis	PD Symptoms
6-OHDA	5-(2-aminoethyl) benzene-1,2,4-triol	Unilateral lesion (midforebrain or striatum) Bilateral lesion (striatum, dorsolateral striatum or midforebrain) Intracerebroventricular injection Intracranial injection Intranigral injection	Striatal dopaminergic neurons ↓. Striatal tyrosine hydroxylase ↓. Nigral tyrosine hydroxylase ↓.	Rotational motor behavior ↑ after injections(Postural asymmetry) (Bilateral injections: akinesia).
MPTP	1-Methyl-4-phenyl-1,2,3,6-tetra hydropyridine	Subcutaneous injection Intraperitoneal injection Intramuscular injection Intranigral injection Intranasal administration Intravenous injection	Dopaminergic neurons ↓. Striatal dopamine levels ↓. MPTP is metabolized into the toxic cation MPP^+^ by the enzyme monoamine oxidase-B of glial cells.	Motor imbalance ↑ in primates (Hypokinesia). Motor disturbance ↑ in acute rodents.
MPP^+^	1-Methyl-4-phenyl pyridinium	Intracerebroventricular Intrastriatal injection		
Paraquat	1,1′-dimethyl-4,4′-bipyridinium dichloride	Subcutaneous injection Intraperitoneal injection Oral administration Aerosol administration Intragastric injection	Striatal tyrosine hydroxylase ↓. α-synuclein ↑.	No clear motor defects, but slightly ↓ ambulatory behaviors, stereotypy and rotational activities.
Rotenone	(2R,6aS,12aS)-1,2,6,6a,12,12 a-hexahydro-2-isopropenyl-8,9-dimethoxychromeno [3,4-b] furo (2,3-h)chromen-6-one	Unilateral (into right striatum or right substantia nigra) Subcutaneous injection Intraperitoneal injection	Dopaminergic neurons ↓ Striatal dopamine ↓. α-synuclein ↑.	Motor disturbances ↑ in rodents.
Permethrin	3-phenoxybenzyl-(1R,S)-cis,trans-3-(2,2-dichlorovinyl)-2,2-dimethylcyclopropane carboxylate	Oral administration	Striatal dopaminergic neurons ↓. α-synuclein ↑.	Motor disturbances ↑. Learning/ memory impairments ↑.

**Table 2:** The dose, lesion type and behavioral evaluating methods in each 6-OHDA-induced PD animal model.

6-OHDA Dose	Animal	Lesion Type	Description of Behavioral Tests Performed	Reference
4 µg/µL/min/rat (in saline-containing 0.02% ascorbic acid)	Sprague Dawley rats	Unilateral injection (right striatum)	0.5 mg/kg apomorphine-induced rotation: Second, 4th, 6th, and 8th weeks post-grafting.	[85]
0.3, 0.7, 1, or 3.6 µg/µL. (in 0.02% ice-cold ascorbate/saline solution and used within 3 h)	Male Rgs5^gfp/+^ reporter mice	Unilateral injection (midforebrain)	Corridor test: three weeks after the lesion. Cylinder test: five weeks after the lesion. Stepping test: five weeks after the lesion. Rotational tests: after the stepping test (fifth week) Spontaneous rotation Apomorphine-induced rotation (0.1 mg/kg, subcutaneously) Amphetamine-induced rotation (5 mg/kg, intraperitoneally).	[86]
5 µg in 5 µL at 1 µL/min (2 mg/mL 6-OHDA) injection rate (in 0.01% ascorbic acid prepared in saline and protected from light and stored at 20 °C)	Male Sprague Dawley rats	Unilateral injection (right midforebrain)	Apomorphine-induced (0.05 mg/kg, subcutaneously) rotation: during the 6th week after the lesion. Amphetamine-induced (3 mg/kg, intraperitoneally) rotation: on the 10th–12th day after the lesion. Rotarod test: during the 2nd and 4th week after the lesion. Open-field test: during the 5th week after the lesion.	[87]
6 µg in 3 µL at 0.3 µL/min injection rate (in 3 µL of 0.9% saline solution containing 0.008% L-ascorbic acid)	Male Wistar rats	Unilateral injection (left midforebrain)	Adjusting step test: on the 5th day after the lesion (Forelimb use asymmetry test).	[88]
40 µg/site (in saline containing 0.1% ascorbic acid)	Male Swiss mice	Intracerebroventricular	Single pellet reaching task: on the 7th day after the lesion. Grid test: on the 7th day after the lesion. Open-field test: on the 8th day after the lesion.	[89]
5/10/15 µg in 4 µL (in saline)	Male Sprague Dawley rats	Intracranial injection (right midforebrain)	Rat motor function assessment: a rotarod machine with automatic timers and falling sensors was used to assess the motor function of the rats.	[90]
6 µg in 1 µL at 0.33 µL/min injection rate for 3 min (6 µg in 1 µL of artificial cerebrospinal fluid, supplemented with 0.2% ascorbic acid)	Male Wistar rats	Bilateral intranigral injection	Open-field test: on the 1st and 21st day after the lesion. Elevated plus maze test: on the 21st day after the lesion. Contextual fear conditioning test: on the 24th day after the lesion.	[91]
6 µg in three sites at 0.5 µL/min injection rate (in saline with 0.02% ascorbic acid)	Male Wistar rats	Intrastriatal injection (left midforebrain)	Amphetamine-induced (2.5 mg/kg, subcutaneously) rotation: during the 1st–2nd week and the 8th–14th week after the lesion.	[92]
100, 300, 500, 700, or 1000 µg in 4 µL (100 µg/day 1st–10th day) at 1 µL/min injection rate (in 0.02% ascorbate solution)	Male Sprague Dawley rats	Intracerebroventricular	Catalepsy grid test: first set: before 6-OHDA or sham lesions, 2 h and 24 h after each daily infusion and daily until six days’ after the lesion; second set: same as in the first set and also on the 11th, 18th, 25th, and 32nd day after the lesion.	[93]
7 µg/µL (in 0.9% saline solution containing 0.02% ascorbic acid)	Male Sprague Dawley rats	Unilateral injection (right midforebrain)	Apomorphine-induced (0.5 mg/kg, intraperitoneally) rotation: during 1st, 3rd, and 5th week after the lesion.	[94]
0.5, 1, 2, or 4 µg/µL in 1 µL at 0.5 µL/min injection rate (in saline with 0.1% ascorbic acid)	C57BL/6 mice	Unilateral injection (right midforebrain)	Cylinder test: during 3rd week after the lesion. Electrophysiological recordings: during 3rd week after the lesion.	[95]
6, 12, or 24 µg in 0.5 µL (in 0.3% ascorbic acid in saline)	*Proechimys* (Spiny rats)	Unilateral injection (right striatum)	Cylinder test: baseline, 5th, 10th, and 15th day after the lesion. Apomorphine-induced rotation (0.5 mg/kg, subcutaneously): baseline 5th, 10th and 15th day after the lesion.	[96]
4 µL (4 µg/µL) into two Sites (in isotonic saline containing 0.2 mg/mL of ascorbic acid)	Male Wistar rats	Unilateral injection (midforebrain)	Apomorphine-induced (0.5 mg/kg, intraperitoneally) rotation: before 6-OHDA and in 3rd week after the lesion.	[97]
6, 10, or 16 µg in 2 µL at 0.1 µL/min (in 0.02% ascorbic acid)	Male Wistar rats	Unilateral injection (left substantia nigra)	Turning behavior test: on the 12th day after the lesion. Cylinder test: five days before, on the 15th and 30th day after the lesion. Measurement of tactile allodynia: on the 18th–28th day after the lesion. Measurement of mechanical hyperalgesia: on the 18th and 28th day after the lesion.	[98]
8 µg in 4 µL (in saline and ascorbic acid)	Male Sprague Dawley rats	Unilateral injection (substantia nigra)	Rotarod test: during 1st, 2nd, and 3rd week after the lesion. Open-field test: during the 5th week after the lesion. Grid test: baseline and 5th week after the lesion.	[99]
20 µg 6-OHDA in 4 µl at 1 µL/min injection rate (in saline and ascorbic acid)	Charles Foster strain of male albino rats	Unilateral injection (striatum)	Apomorphine-induced (1 mg/kg, intraperitoneally) rotation: baseline, 7th, 14th, 21st and 28th day after the lesion. Open-field test: baseline, 7th, 14th, 21st, and 28th day after the lesion. Rotarod test: baseline, 7th, 14th, 21st, and 28th day after the lesion. Grip strength test: baseline, 7th, 14th, 21st, and 28th day after the lesion. Bar catalepsy test: baseline, 7th, 14th, 21st, and 28th day after the lesion.	[100]
6 µg in 1 µL (in saline and ascorbic acid)	Male Wistar rats	Unilateral injection (Intracerebral infusion)	Apomorphine-induced (3 mg/kg, subcutaneously) rotation: rotational (circling) behavior test performed on the 14th day and 1-h after the last *Spirulina platensis* administration.	[101]
5, 10, or 14 µg/µL (in saline and ascorbic acid)	Male C57BL/6 mice	Bilateral administration (into locus coeruleus)	Forced swim test: three weeks after the lesion. Sucrose consumption: four days before (pretest) and 3rd week after the lesion.	[102]
8, 12, or 16 µg/4 µL at 0.5 mL/min injection rate (in saline and ascorbic acid)	Male Wistar Han rats	Unilateral injection (midforebrain)	Sucrose preference test: first and 3rd week after the lesion. Apomorphine-induced (0.25 mg/kg, subcutaneously) rotation: Second and 4th week after the lesion.	[103]
8 µg/2 µL/rat at 0.2 µL/min injection rate (in saline and ascorbic acid)	Male Wistar rats	Intranigral injection	Catalepsy assay test (bar test): on the 21st, 22nd, and 26th day after the lesion. Rotarod test: on the 21st, 22nd, and 26th day after the lesion.	[104]
8 µg/2 µL/rat at 0.2 µL/min injection rate (in saline and ascorbic acid)	Male Wistar rats	Unilateral injection	Catalepsy assay test (bar test): on the 26th day after the lesion. Rotarod test: on the 26th day after the lesion.	[105]
17.5 µg in 2.5 µL at 0.5 µL/min injection rate (saline and ascorbic acid)	Female Sprague Dawley rats	Unilateral injection (right midforebrain)	Cylinder test: Third week after the lesion. Ratings of axial, limbs and orolingual abnormal involuntary movements: Third–6th week after the lesion.	[106]
20 µg in 3 µL at 1 µL/min injection rate (in saline and ascorbic acid)	Male Wistar rats	Bilaterally into dorsolateral striatum	Open-field test: on the 7th and 35th day after the lesion. Rotarod test: on the 7th, 21st, and 35th day after the lesion.	[107]
5 µg in 1 µL at 0.5 µL/min injection rate (in saline and ascorbic acid)	Male mice (CD-1)	Intrastriatal (right striatum)	Rotarod test: on the 12th and 14th day after the lesion.	[108]
8 µg/2 µL (in saline and ascorbic acid)	Male Wistar rats	Unilateral injection	Rotational behavior: tests for the amphetamine (5 mg/kg, subcutaneously) and apomorphine-induced (0.25–0.75 mg/kg, subcutaneously) rotations: during the 1st, 3rd, and 6th week after the lesion.	[109]
8 µg/ rat (in saline and ascorbic acid)	Male Sprague Dawley rats	Unilateral injection (left midforebrain)	Apomorphine-induced (0.5 mg/kg, subcutaneously) rotation: on the 7th, 14th, 21st, and 28th day after the lesion. Open-field tests: on the 4th day after the lesion.	[110]
20 µg/4 µL at 0.5 µL/min injection rate (in saline and ascorbic acid)	Male Sprague Dawley rats	Unilateral injection (midforebrain)	Apomorphine-induced (0.2 mg/kg, subcutaneously) rotation: on the -2nd and 28th day after the lesion.	[111]
20 µg in 4 µL (2 µL/site) at 0.2 µL/min injection rate (in saline and ascorbic acid)	Male Sprague Dawley rats	Unilateral injection (left striatum)	Apomorphine-induced (0.5 mg/kg, subcutaneously) rotation: baseline, 2nd, 3rd, 4th and 5th week after the lesion. Rotarod test: baseline, 2nd, 3rd, 4th, and 5th week after the lesion. Cylinder test: baseline, 2nd, 3rd, 4th, and 5th week after the lesion. Open-field test: baseline, 2nd, 3rd, 4th, and 5th week after the lesion.	[112]
16 µg/4 µL/rat (in 0.2% ascorbic acid)	Female Sprague Dawley rats	Unilateral injection (right midforebrain)	Axial, limbs and orolingual and abnormal involuntary movements: the third week after the lesion. Forelimb functional test: on the 5th, 13th, and 20th day after the lesion.	[113]
8 µg/3 µL (in saline)	Male Sprague Dawley rats	Intracranial stereotaxic administration (left substantia nigra)	Elevated body swing testing: on the 14th, 28th, 42nd, and 56th day after the lesion. Beam walk: on the 28th, 42nd, and 56th day after the lesion. Apomorphine-induced (0.3 mg/kg, subcutaneously) contralateral rotation: on the 14th, 28th, 42nd, and 56th day after the lesion. Rotarod test: on the 28th, 42nd, and 56th day after the lesion.	[114]
16 µg (8 µL/site) at 0.5 µL/min injection rate (in saline and ascorbic acid)	Male Sprague Dawley rats	Unilateral injection (right midforebrain)	Abnormal involuntary movement: on the 2nd, 9th, 11th, 18th, and 21st day after levodopa. Rotational response duration: on the 2nd, 9th, 11th, 18th, and 21st day after levodopa. Forepaw adjusting step: on the 2nd, 9th, 11th, 18th, and 21st day after levodopa.	[115]
24 µg/4 µL (in 0.05% ascorbate saline)	Female Wistar rats	Unilateral injection	The limb-use asymmetry (cylinder) test: three weeks after the lesion. Rotarod test: on the 17th day. Cat Walk test: on the 16th day. Sucrose preference test: on the 16th and 17th day. Elevated plus maze test: on the 16th day. Novel object recognition test: on the 17th day. Abnormal involuntary movement score: on the 1st, 4th, 7th, 10th, 13th, and 15th day after levodopa.	[116]
5, 10, or 20 µg/2 µL/hemisphere at 1 µL/min injection rate (in saline and ascorbic acid)	Male Wistar rats	Bilateral injection	Open-field test: on the 7th, 21st, or 42nd day after the lesion. Rotarod test: on the 7th, 21st, or 42nd day after the lesion. Olfactory discrimination test: on the 7th, 21st, or 42nd day after the lesion. Object recognition task: on the 7th, 21st, or 42nd day after the lesion. Step-down inhibitory avoidance task: on the 7th, 21st, or 42nd day after the lesion. Forced swimming test: on the 7th, 21st, or 42nd day after the lesion. Elevated plus maze test: on the 7th, 21st, or 42nd day after the lesion. Sucrose preference test: on the 7th, 21st, or 42nd day after the lesion.	[117]
8 µg/4 µL at 0.5 µL/min injection rate (in saline and ascorbic acid)	Male Sprague Dawley rats	Unilateral injection (right substantia nigra)	Apomorphine-induced (0.5 mg/kg, intraperitoneally) rotation: third and 5th week after the lesion.	[118]
10 µg/2 µL at 0.5 µL/min injection rate (in saline and ascorbic acid)	Male CD1 wild-type mice	Intrastriatal injection	Cylinder test: on the 7th day after the lesion. Abnormal involuntary movements rating test: on the 20th and 28th day after the lesion. Gait test: on the -3rd, 21st, and 29th day after the lesion.	[119]
6 µg/2.5 µL (in saline and ascorbic acid)	Male albino Wistar rats	Intrastriatal injection	Apomorphine-induced rotation (1 mg/kg, intraperitoneally): on the 15th day after the lesion. Pole test: on the 15th day after the lesion. Catalepsy test: on the 15th day after the lesion. Beam walking test: on the 15th day after the lesion. Rotarod test: on the 5th, 10th, and 15th day after the lesion. Open-field test: on the 15th day after the lesion.	[120]
12 µg/6 µL at 1 µL/min injection rate (in saline and ascorbic acid)	Male Sprague Dawley rats	Unilateral intracerebral injection	Apomorphine-induced (0.5 mg/kg, intraperitoneally) rotation: before 6-OHDA lesion and three weeks after the safflower flavonoid extract treatment.	[121]
20 µg/3 µL at 1 µL/min injection rate (in 0.02% ascorbic acid)	Male Sprague Dawley rats	Unilateral injection (right striatum)	Cylinder test: baseline and 28th day after the lesion. Apomorphine-induced (0.5 mg/kg, intraperitoneally) rotation: baseline and 28th day after the lesion.	[122]
6 µg/2 µL (in saline and ascorbic acid)	Male Wistar rats	Intranigral injection (right substantia nigra)	Apomorphine-induced rotation: on the 1st, 7th, and 14th day after the lesion. Morris water-maze test: on the 1st, 7th, and 14th day after the lesion.	[123]
4 µg/2 µL (2 µg/µL for 2 sites) (in saline and ascorbic acid)	Male C57BL/6 mice	Intrastriatal injection (right striatum)	Apomorphine-induced (0.1 mg/kg, subcutaneously) rotation: two weeks after the lesion.	[124]
6 µg/2 µL (in saline and ascorbic acid)	Male C57BL/6 mice	Intrastriatal injection (right striatum)	Apomorphine-induced (0.1 mg/kg, subcutaneously) rotation: on the 7th, 14th, and 21st day after the lesion.	[125]

**Table 3:** The dose, lesion type and behavioral evaluating methods in each MPTP-induced PD animal model.

MPTP Dose	Animal	Lesion Type	Description of Behavioral Tests Performed	Reference
2 mg/kg/injection for five days, a total of 10 mg/kg(in saline)	Common marmosets *(Callithrix jacchus)*	Subcutaneously	Rating of motor disability: during the acclimatization period and once every 10 min after drug treatment for 3 h using a motor disability rating scale. Locomotor activity: assessed the number of beam interruptions accumulated every 10 min within 3 h of drug treatment.	[132]
10 mg/kg/injection for three days, a total of 30 mg/kg (in saline)	Male C57BL6 mice	Intraperitoneally	Cylinder test: on the 11th day. Rotarod test: on the 1st and 11th day.	[13]
10 mg/kg/injection for three days, a total of 30 mg/kg (in saline)	Male C57BL6 mice	Intraperitoneally	Rotarod test: before electroacupuncture and on the 8th day after MPTP injection.	[133]
20 mg/kg/injection four times/day at 2-h intervals, a total of 80 mg/kg (in saline)	Male C57BL/6 J mice	Intraperitoneally	Pole test: on the 6th day after MPTP injection.	[134]
15 mg/kg/injection for seven days, a total of 105 mg/kg (in saline)	Female BALB/c mice	Intraperitoneally	Cylinder test: on the 8th day. Open-field locomotion activity test: on the 8th day.	[135]
15 mg/kg/injection for seven days, a total of 105 mg/kg (in saline)	Female BALB/c mice	Intraperitoneally	Open-field locomotion activity test: on the 8th day. Rearing test: on the 9th day.	[136]
25 mg/kg/injection at 3.5-day intervals for five weeks, total 125 mg/kg (in saline)	Male C57BL/6 J mice	Subcutaneously	Open-field test: on the 61st day. Static bar test: on the 53rd day. Horizontal bar test: on the 54th day. Olfactory discrimination tests: on the 11th, 87th, and 90th day. Nesting behavior: on the 75th day.	[137]
0.1 mg/nostril (2–three doses, baseline and 7th day) (in 10% *w/v* ethanol and 0.9% saline)	Male albino Wistar rats	Intranasal administration	Olfactory discrimination: on the 7th, 14th, and 21st day. Forced swim test: on the 7th, 14th, and 21st day. Rotarod test: on the 7th, 14th, and 21st day. Locomotor activity: on the 7th, 14th, and 21st day.	[138]
20 mg/kg/injection at 2-h intervals; a total 40 mg/kg (in saline)	Male C57Bl/6 mice	Intraperitoneally	Rotarod test: on the 6th day after MPTP injection.	[139]
15 mg/kg/injection four times/day at 2-h intervals; total 60 mg (0.1% ethanol in saline)	Female C57BL/6 mice	Intraperitoneally	Rotarod test: on the 9th day after MPTP injection.	[140]
30 mg/kg/day for eight days (in saline)	Male C57BL/6 mice	Intraperitoneally	Spontaneous motor activity test: on the 7th day after MPTP injection. Rotarod test: after the spontaneous motor activity test.	[141]
0.2 mg/kg/day, until Parkinsonian motor signs appeared (in saline)	Captive-bred monkeys (*M. fascicularis*)	Intravenous	Assessment of motor behaviors: after MPTP administration. Cognitive task performances.	[142]
30 mg/kg/day for five days, a total of 150 mg/kg (in saline)	Male C57BL/6 J mice	Intraperitoneally	Pole test: on the 1st, 5th, 10th, 15th, and 20th day after MPTP injection.	[143]
30 mg/kg/day for five days, a total of 150 mg/kg (in saline)	Male C57BL/6 mice	Intraperitoneally	Gait analysis (catwalk system): performed 90 min after levodopa administration (on the 21st day) before sacrifice.	[144]
0.2 mg/kg/day at 1 mL/min injection rate until the appearance of typical PD (in saline)	Cynomolgus monkeys (*M. fascicularis*)	Intraperitoneally	Video recording and clinical rating: during and after MPTP injection. Measurement of overall home-cage activity level: regularly monitored in the home cage for 8 h/day from the beginning of MPTP injection.	[145]
0.2 mg/kg (in saline)	Adult female cynomolgus monkeys (*M. fascicularis*)	Intramuscularly	Video recording: once per week for a total of 4 h recorded for each monkey. Parkinsonian behavior assessment: video recordings 5 min periods, every 60 min (total 240 min) using the Kurlan scale. Global activity: video recordings 240 min. for each experimental animal using video global activity analysis system software.	[146]
0.5 mg/kg/week for five weeks, a total of 2.5 mg/kg (in saline)	Marmoset monkeys (*C. jacchus*)	Subcutaneously	Observational clinical signs: 2–three times during the week. Human threat test: by the eye contact from 30–50 cm from the cage front and for a 2-min test period. Home cage activity: over the total experimental period. Hand–eye coordination: baseline, during MPTP exposure (last three weeks) and in the recovery period (last three weeks). Hourglass test: baseline, MPTP exposure (last three weeks) and in the recovery period (last three weeks). Tower test: baseline, MPTP exposure (last three weeks), in the recovery period (last three weeks). Bungalow test: baseline, MPTP exposure (last three weeks) and in the recovery period (last three weeks).	[147]
10 mg/mL, a subcutaneously implanted device on the right side of the back	Female Gottingen minipigs	Subcutaneously	Behavioral score: baseline, 1st, 2nd, 2.5, 4th, 5th, 7th, and 10th–11th week. Gait analysis: after the 4th and 11th week of MPTP administration.	[148]
10, 20, or 30 mg/kg/injection four times/day at 2-h intervals (in saline)	Male C57BL/6 N mouse	Intraperitoneally	Rotarod test: on the 3rd day. Pole test: after MPTP injection.	[149]
15 mg/kg/day for four days, a total of 60 mg/kg (in saline)	Male C57BL/6 mice	Intraperitoneally	Rotarod test: on the 31st and 35th day.	[150]
2 mg/kg for two days and then 1 mg/kg for next three days, total 7 mg/kg	Male common marmoset (*C. jacchus*)	Subcutaneously	Tower test: once per day for one week and then weekly until the end of the experimental period.	[151]
2.5 mg/kg/injection twice per day, a total of 5 mg (in saline)	Male mice (G2019S-LRRK2 mutation)	Subcutaneously	Open-field test: at 6 and 12 months’ age. Rotarod testing: on the 5th–7th day after MPTP injection.	[152]
20 mg/kg/injection four times/day at 2-h intervals, a total of 80 mg/kg (in saline)	Male C57BL/6 mice	Intraperitoneally	Locomotor activity: baseline, 2nd, 4th, and 8th day. Rotarod test: baseline, 2nd, 4th, and 8th day.	[153]
25 mg/kg/day for four days, a total of 100 mg/kg (in H_2_O)	Male C57Bl/6 J mice	Intraperitoneally	Open-field test: three days after the last MPTP injection (on the 7th day). Pole test: three days after the last MPTP injection (on the 7th day).	[154]
30 mg/kg/injection for four days, a total of 120 mg/kg (in saline)	Male C57BL/6 mice	Intraperitoneally	Open-field test: 7 days after the last MPTP injection. Narrow beam test: 7 days after the last MPTP injection. Hang test: 7 days after the last MPTP injection. Catalepsy test: 7 days after the last MPTP injection.	[155]
30 mg/kg/day for five days, a total of 150 mg/kg (in saline)	Male C57BL/6 J mice	Intraperitoneally	Rotarod test: on the 6th, 9th, and 12th day after MPTP injection. Pole test: on the 6th and 12th day after MPTP injection. Open-field test: on the 12th day after MPTP injection.	[156]

**Table 4:** The dose, lesion type and behavioral evaluating methods in each MPP^+^-induced PD animal model.

MPP^+^ Dose	Animal	Lesion Type	Description of Behavioral Tests Performed	Reference
1.8 µg/site (in saline)	Male C57BL6 mice	Unilateral (intracerebroventricular) injection	Tail suspension test: 24 h after MPP^+^ administration. Open-field test: ten minutes after the tail suspension test. Splash test: after the open-field test.	[161]
1.8–18 µg (in saline)	Male C57BL6 mice	Unilateral (intracerebroventricular) injection	Forced swim test: on the 1st, 7th, and 30th or 100th day. Tail suspension test: on the 1st, 7th, and 30th or 100th day. Splash test: on the 1st, 7th, and 30th or 100th day. Elevated plus maze test: on the 1st, 7th, and 30th or 100th day. Step-down inhibitory avoidance test: on the 1st, 7th, and 30th or 100th day. Rectangular open-field test: on the 1st, 7th, and 30th or 100th day. Circular open-field test: on the 1st, 7th, and 30th or 100th day. Rotarod test: on the 1st, 7th, and 30th or 100th day.	[162]
8 µg/site (in saline)	Sprague Dawley rats	Unilateral injection	Apomorphine-induced rotation (5 mg/kg, intraperitoneally) and locomotor activity: 8 days after MPP^+^ administration.	[133]
10 µg/site (in 8 µL saline)	Male Wistar rats (strain NIH)	Unilateral (intrastriatal) injection	Apomorphine-induced rotational behavior (1 mg/kg apomorphine, subcutaneously): 6 days after MPP^+^ administration.	[163]
10 µg/site in (in 8 µL saline)	Male Wistar rats	Unilateral (intrastriatal) injection	Apomorphine-induced rotational behavior (1 mg/kg apomorphine, subcutaneously): 6 days after MPP^+^ administration.	[164]
15 µg/site (in 8 µL saline)	Male Wistar rats	Unilateral (intrastriatal) injection	Apomorphine-induced rotational behavior (1 mg/kg apomorphine, subcutaneously): 6 days after MPP^+^ administration.	[165]
15 µg/site (in 8 µL saline)	Male Wistar rats (NIH strain)	Unilateral (intrastriatal) injection	Apomorphine-induced rotational behavior (1 mg/kg apomorphine, subcutaneously): 6 days after MPP^+^ administration.	[166]
30 µg/site (in 4 µL saline)	Sprague Dawley rats	Unilateral (intrastriatal) injection	Open-field test: on the 3rd day after intracerebroventricular injection of transforming growth factor-β1. Rotarod test: on the 3rd day after intracerebroventricular injection of transforming growth factor-β1. Apomorphine-induced rotational behavior (1 mg/kg, intraperitoneally): on the 3rd day after intracerebroventricular injection of transforming growth factor-β1.	[167]

**Table 5:** The dose, lesion type, and behavioral evaluating methods in each paraquat-induced PD animal model.

Paraquat Dose	Animal	Lesion Type	Description of Behavioral Tests Performed	Reference
0.02 or 0.2 mg/kg postnatal day 10 and 11 (in H_2_O and sonicated with a 20% fat emulsion vehicle)	Male C57Bl/6 mice	Oral administration	Spontaneous behavior: at 2 months of age. Radial arm maze test: at 3 months of age.	[176]
1 or 10 mg/kg, two times/week for three weeks (on the 1st, 5th, 8th, 12th, 15th, and 19th days) (in saline)	Male C57/BL6 mice	Intraperitoneally	Home cage locomotor activity: baseline, 8th, 15th, and 19th day. Sucrose preference test: baseline, 2nd, 9th, and 20th day. Spontaneous alternation behavior Y-maze test: on the 21st day. Open-field test: on the 22nd day. Elevated plus maze test: on the 22nd day. Forced swim test: on the 24th day.	[177]
1 mg/kg/day for seven days, a total of 7 mg/kg (in saline)	Male Sprague Dawley rats	Oral administration	Vibrissae-evoked forelimb placement test (vibrissae test): baseline, 2nd and 4th week. Stepping test: baseline, 2nd and 4th week.	[178]
10 mg/kg twice per week for three weeks, a total of 60 mg/kg (in saline)	Male C57BL6/J mice	Intraperitoneally	Home cage locomotor activity: baseline, 1st, 6th, 13th, 20th, 22nd, 29th, and 40th day. Sucrose preference test: baseline, 1st, 7th, 14th, 21st, 23rd, 31st, and 41st day. Spontaneous alternation behavior Y-maze test: on the 19th and 42nd day. Elevated plus maze test: on the 21st and 43rd day. Rotarod test: on the 41st day. Forced swim test: on the 44th day.	[179]
2.5 mg/kg/day for four weeks; 0.25 µL/h injection rate (in saline)	Male Wistar rats	Subcutaneously (chronic exposure using osmotic minipumps implanted slightly posterior back to the shoulder blades)	Rotarod test: eight weeks after the implantation of the minipumps. Open-field test: eight weeks after the implantation of the minipumps.	[180]
10–20 mg/kg (200 g/L gramoxone, from 1st day of pregnancy (G0) to G21)	Male and female Swiss mice	Oral administration	Negative geotaxis test: prenatal exposure, on the 5th, 7th, and 9th day. Surface righting reflex test: prenatal exposure, on the 5th, 7th, and 9th day. Cliff avoidance test: prenatal exposure, on the 5th, 7th, and 9th day. Rotarod test: prenatal exposure, on the 23rd, 24th, and 25th. Paraquat effect on adult behavior: all animals were tested on the postnatal 60th day for the open-field test, elevated plus maze test and novel object recognition).	[181]
27 or 54 mg/m^3^ eight times (on the 1st, 3rd, 5th, 7th, 9th, 13th, and 15th day), each time 30 min, for 16 days.	Sprague Dawley rats	Aerosol administration	Morris water-maze test: on the 1st, 2nd, 3rd, 4th, and 5th day. Passive avoidance test: latency to enter the darkroom, 3, 24, and 48 h.	[182]
5 mg/kg four times (on the 2nd, 4th, 6th, and 7th day; a total of 20 mg/kg) (in saline)	Male Swiss mice	Intraperitoneally	Open-field test: two days after the last paraquat administration. Rotarod test: sixty days after the last paraquat administration. Neurological Severity Score: on the 2nd, 15th, 30th, and 60th day after the last paraquat administration. Social recognition: thirty-eight days after the last paraquat administration.	[183]
7 mg/kg with two-day intervals for a total of six doses; a total of 42 mg/kg (in saline)	C57Bl6/J (p16-3MR) mice	Intraperitoneally	Cylinder test: before the sacrifice.	[184]

**Table 6:** The dose, lesion type, and behavioral evaluating methods in each rotenone-induced PD animal model.

Rotenone Dose	Animal	Lesion Type	Description of Behavioral Tests Performed	Reference
0.5 mg/mL at 1 mL/kg/day for 35 days (in sunflower oil)	Sprague Dawley rat pups	Intraperitoneally	Rotarod test: on the 15th and 45th day after the last rotenone injection. Open-field test: on the 15th and 45th day after the last rotenone injection.	[190]
1.5, 2, or 2.5 mg/kg day for five weeks (in sunflower oil)	Male Wistar rats	Subcutaneously	Rearing test: after five weeks of rotenone injection. Catalepsy test: grid and bar test after five weeks of rotenone injection. Rotarod test: after five weeks of rotenone injection. Locomotor activity: after five weeks of rotenone injection.	[191]
2 mg/kg (in chloroform and 0.5% carboxymethyl cellulose solution)	Male Sprague Dawley rats	Subcutaneously	Grip strength: baseline, 10th and 20th day. Y-maze test: baseline, 10th and 20th day.	[192]
2 mg/kg/day for 35 days (in sunflower oil at 2 mg/mL)	Male albino Wistar rats	Subcutaneously	Rearing behavior: on the 36th day. Rotarod test: on the 36th day.	[193]
2 mg/kg/day for 35 days (2 mg/mL of rotenone in 98% sunflower oil and 2% dimethyl sulfoxide)	Male albino Wistar rats	Subcutaneously	Cylinder test: on the 36th day.	[194]
2.5 mg/kg/day for 21 days (in sunflower oil)	Adult male Wistar albino rats	Intraperitoneally	Cylinder test: baseline, 1st, 2nd, 3rd, 4th, 5th, and 6th week.	[195]
2.5 mg/kg/day for 10 or 45 days (in sunflower oil)	Male albino Wistar rats	Intraperitoneally	Open-field test: on the 1st, 7th, 14th, 21st, and 28th day after the last rotenone injection. Catalepsy test: on the 46th day. Akinesia test: on the 46th day. Forced swim test: 29 days after the last rotenone injection (39th experimental day). Sucrose preference test: on the 14th and 21st day after rotenone injection (24th and 31st experimental days, respectively). Elevated plus maze test: baseline and 46th day.	[196]
3 mg/kg/day for 10 days (in 2% dimethyl sulfoxide and 98% polyethylene glycol 400)	Male albino rats	Subcutaneously	Cylindrical test: before sacrifice. Catalepsy test: before sacrifice.	[197]
4 µg /site; a total of 12 µg for three sites (in 2 μL of dimethyl sulfoxide and saline)	Male Sprague Dawley rats	Unilateral injection (into right striatum)	Postural instability test: two weeks after rotenone injection.	[198]

**Table 7:** Dose, lesion type, and behavioral evaluating methods in each permethrin-induced PD animal model.

Permethrin Dose	Animal	Lesion Type	Description of Behavioral Tests Performed	Reference
0.05 mg/kg/day (in drinking water)	Male and female C57BL/6 mice	Oral administration	Open-field test: 10th–11th weeks old. Light/dark transition test: 10th–11th weeks old. Contextual/cued fear conditioning test: 10th–11th weeks old.	[205]
34 mg/kg/daily from postnatal day 6th–21st (in corn oil)	Male and female Wistar rats	Oral administration	Rotarod test: on the postnatal 50th, 100th, and 150th day. Footprint and beam walking: on the postnatal 50th, 100th and 150th day. T-maze test: on the postnatal 50th, 100th, and 150th day.	[206]
